# Identification of independent association signals and putative functional variants for breast cancer risk through fine-scale mapping of the 12p11 locus

**DOI:** 10.1186/s13058-016-0718-0

**Published:** 2016-06-21

**Authors:** Chenjie Zeng, Xingyi Guo, Jirong Long, Karoline B. Kuchenbaecker, Arnaud Droit, Kyriaki Michailidou, Maya Ghoussaini, Siddhartha Kar, Adam Freeman, John L. Hopper, Roger L. Milne, Manjeet K. Bolla, Qin Wang, Joe Dennis, Simona Agata, Shahana Ahmed, Kristiina Aittomäki, Irene L. Andrulis, Hoda Anton-Culver, Natalia N. Antonenkova, Adalgeir Arason, Volker Arndt, Banu K. Arun, Brita Arver, Francois Bacot, Daniel Barrowdale, Caroline Baynes, Alicia Beeghly-Fadiel, Javier Benitez, Marina Bermisheva, Carl Blomqvist, William J. Blot, Natalia V. Bogdanova, Stig E. Bojesen, Bernardo Bonanni, Anne-Lise Borresen-Dale, Judith S. Brand, Hiltrud Brauch, Paul Brennan, Hermann Brenner, Annegien Broeks, Thomas Brüning, Barbara Burwinkel, Saundra S. Buys, Qiuyin Cai, Trinidad Caldes, Ian Campbell, Jane Carpenter, Jenny Chang-Claude, Ji-Yeob Choi, Kathleen B. M. Claes, Christine Clarke, Angela Cox, Simon S. Cross, Kamila Czene, Mary B. Daly, Miguel de la Hoya, Kim De Leeneer, Peter Devilee, Orland Diez, Susan M. Domchek, Michele Doody, Cecilia M. Dorfling, Thilo Dörk, Isabel dos-Santos-Silva, Martine Dumont, Miriam Dwek, Bernd Dworniczak, Kathleen Egan, Ursula Eilber, Zakaria Einbeigi, Bent Ejlertsen, Steve Ellis, Debra Frost, Fiona Lalloo, on behalf of EMBRACE, Peter A. Fasching, Jonine Figueroa, Henrik Flyger, Michael Friedlander, Eitan Friedman, Gaetana Gambino, Yu-Tang Gao, Judy Garber, Montserrat García-Closas, Andrea Gehrig, Francesca Damiola, Fabienne Lesueur, Sylvie Mazoyer, Dominique Stoppa-Lyonnet, behalf of GEMO Study Collaborators, Graham G. Giles, Andrew K. Godwin, David E. Goldgar, Anna González-Neira, Mark H. Greene, Pascal Guénel, Lothar Haeberle, Christopher A. Haiman, Emily Hallberg, Ute Hamann, Thomas V. O. Hansen, Steven Hart, Jaana M. Hartikainen, Mikael Hartman, Norhashimah Hassan, Sue Healey, Frans B. L. Hogervorst, Senno Verhoef, on behalf of HEBON, Carolyn B. Hendricks, Peter Hillemanns, Antoinette Hollestelle, Peter J. Hulick, David J. Hunter, Evgeny N. Imyanitov, Claudine Isaacs, Hidemi Ito, Anna Jakubowska, Ramunas Janavicius, Katarzyna Jaworska-Bieniek, Uffe Birk Jensen, Esther M. John, Charles Joly Beauparlant, Michael Jones, Maria Kabisch, Daehee Kang, Beth Y. Karlan, Saila Kauppila, Michael J. Kerin, Sofia Khan, Elza Khusnutdinova, Julia A. Knight, Irene Konstantopoulou, Peter Kraft, Ava Kwong, Yael Laitman, Diether Lambrechts, Conxi Lazaro, Loic Le Marchand, Chuen Neng Lee, Min Hyuk Lee, Jenny Lester, Jingmei Li, Annelie Liljegren, Annika Lindblom, Artitaya Lophatananon, Jan Lubinski, Phuong L. Mai, Arto Mannermaa, Siranoush Manoukian, Sara Margolin, Frederik Marme, Keitaro Matsuo, Lesley McGuffog, Alfons Meindl, Florence Menegaux, Marco Montagna, Kenneth Muir, Anna Marie Mulligan, Katherine L. Nathanson, Susan L. Neuhausen, Heli Nevanlinna, Polly A. Newcomb, Silje Nord, Robert L. Nussbaum, Kenneth Offit, Edith Olah, Olufunmilayo I. Olopade, Curtis Olswold, Ana Osorio, Laura Papi, Tjoung-Won Park-Simon, Ylva Paulsson-Karlsson, Stephanie Peeters, Bernard Peissel, Paolo Peterlongo, Julian Peto, Georg Pfeiler, Catherine M. Phelan, Nadege Presneau, Paolo Radice, Nazneen Rahman, Susan J. Ramus, Muhammad Usman Rashid, Gad Rennert, Kerstin Rhiem, Anja Rudolph, Ritu Salani, Suleeporn Sangrajrang, Elinor J. Sawyer, Marjanka K Schmidt, Rita K. Schmutzler, Minouk J. Schoemaker, Peter Schürmann, Caroline Seynaeve, Chen-Yang Shen, Martha J. Shrubsole, Xiao-Ou Shu, Alice Sigurdson, Christian F. Singer, Susan Slager, Penny Soucy, Melissa Southey, Doris Steinemann, Anthony Swerdlow, Csilla I. Szabo, Sandrine Tchatchou, Manuel R. Teixeira, Soo H. Teo, Mary Beth Terry, Daniel C. Tessier, Alex Teulé, Mads Thomassen, Laima Tihomirova, Marc Tischkowitz, Amanda E. Toland, Nadine Tung, Clare Turnbull, Ans M. W. van den Ouweland, Elizabeth J. van Rensburg, David ven den Berg, Joseph Vijai, Shan Wang-Gohrke, Jeffrey N. Weitzel, Alice S. Whittemore, Robert Winqvist, Tien Y. Wong, Anna H. Wu, Drakoulis Yannoukakos, Jyh-Cherng Yu, Paul D. P. Pharoah, Per Hall, Georgia Chenevix-Trench, on behalf of KConFab, AOCS Investigators, Alison M. Dunning, Jacques Simard, Fergus J. Couch, Antonis C. Antoniou, Douglas F. Easton, Wei Zheng

**Affiliations:** 1 Division of Epidemiology, Department of Medicine, Vanderbilt-Ingram Cancer Center, Vanderbilt University School of Medicine, 2525 West End Avenue, 8th Floor, Nashville, TN 37203-1738 USA; 2 Centre for Cancer Genetic Epidemiology, Department of Public Health and Primary Care, University of Cambridge, Cambridge, UK; 3 Proteomics Center, CHU de Québec Research Center and Department of Molecular Medicine, Laval University, Quebec, Canada; 4 Centre for Cancer Genetic Epidemiology, Department of Oncology, University of Cambridge, Cambridge, CB1 8RN UK; 5 Department of Surgery, St Vincent’s Hospital, Melbourne, VIC Australia; 6 Centre for Epidemiology and Biostatistics, Melbourne School of Population and Global health, The University of Melbourne, Melbourne, Australia; 7 Cancer Epidemiology Centre, Cancer Council Victoria, Melbourne, Australia; 8 Department of Laboratory Medicine and Pathology, Mayo Clinic, Rochester, MN USA; 9 Immunology and Molecular Oncology Unit, Istituto Oncologico Veneto IOV - IRCCS (Istituto Di Ricovero e Cura a Carattere Scientifico), Padua, Italy; 10 Centre for Cancer Genetic Epidemiology, Department of Oncology, University of Cambridge, Cambridge, UK; 11 Department of Clinical Genetics, Helsinki University Hospital, University of Helsinki, Helsinki, Finland; 12 Lunenfeld-Tanenbaum Research Institute of Mount Sinai Hospital, Toronto, Canada; 13 Department of Molecular Genetics, University of Toronto, Toronto, Canada; 14 Department of Epidemiology, University of California Irvine, Irvine, CA USA; 15 N.N. Alexandrov Research Institute of Oncology and Medical Radiology, Minsk, Belarus; 16 Department of Pathology, Landspitali University Hospital and BMC (Biomedical Centre), Faculty of Medicine, University of Iceland, Reykjavik, Iceland; 17 Division of Clinical Epidemiology and Aging Research, German Cancer Research Center (DKFZ), Heidelberg, Germany; 18 University of Texas MD Anderson Cancer Center, Houston, TX USA; 19 Department of Oncology, Karolinska University Hospital, Stockholm, Sweden; 20 McGill University and Génome Québec Innovation Centre, Montréal, Canada; 21 Human Cancer Genetics Program, Spanish National Cancer Research Centre, Madrid, Spain; 22 Centro de Investigación en Red de Enfermedades Raras, Valencia, Spain; 23 Institute of Biochemistry and Genetics, Ufa Scientific Center of Russian Academy of Sciences, Ufa, Russia; 24 Department of Oncology, Helsinki University Hospital, University of Helsinki, Helsinki, Finland; 25 International Epidemiology Institute, Rockville, MD USA; 26 Department of Radiation Oncology, Hannover Medical School, Hannover, Germany; 27 Copenhagen General Population Study, Herlev Hospital, Copenhagen University Hospital, Herlev, Denmark; 28 Department of Clinical Biochemistry, Herlev Hospital, Copenhagen University Hospital, Herlev, Denmark; 29 Faculty of Health and Medical Sciences, University of Copenhagen, Copenhagen, Denmark; 30 Division of Cancer Prevention and Genetics, Istituto Europeo di Oncologia, Milan, Italy; 31 Department of Genetics, Institute for Cancer Research, Oslo University Hospital Radiumhospitalet, Oslo, Norway; 32 K.G. Jebsen Center for Breast Cancer Research, Institute of Clinical Medicine, Faculty of Medicine, University of Oslo, Oslo, Norway; 33 Department of Medical Epidemiology and Biostatistics, Karolinska Institutet, Stockholm, Sweden; 34 Dr. Margarete Fischer-Bosch-Institute of Clinical Pharmacology, Stuttgart, Germany; 35 University of Tübingen, Tübingen, Germany; 36 German Cancer Consortium (DKTK), German Cancer Research Center (DKFZ), Heidelberg, Germany; 37 International Agency for Research on Cancer, Lyon, France; 38 Division of Preventive Oncology, German Cancer Research Center (DKFZ), Heidelberg, Germany; 39 Netherlands Cancer Institute, Antoni van Leeuwenhoek Hospital, Amsterdam, The Netherlands; 40 Institute for Prevention and Occupational Medicine of the German Social Accident Insurance, Institute of the Ruhr University Bochum, Bochum, Germany; 41 Department of Obstetrics and Gynecology, University of Heidelberg, Heidelberg, Germany; 42 Molecular Epidemiology Group, German Cancer Research Center (DKFZ), Heidelberg, Germany; 43 Department of Medicine, Huntsman Cancer Institute, University of Utah School of Medicine, Salt Lake City, UT USA; 44 Molecular Oncology Laboratory, Hospital Clinico San Carlos, IdISSC (El Instituto de Investigación Sanitaria del Hospital Clínico San Carlos), Madrid, Spain; 45 Cancer Genetics Laboratory, Peter MacCallum Cancer Centre, Melbourne, Australia; 46 Australian Breast Cancer Tissue Bank, Westmead Millennium Institute, University of Sydney, Sydney, Australia; 47 Division of Cancer Epidemiology, German Cancer Research Center (DKFZ), Heidelberg, Germany; 48 University Cancer Center Hamburg (UCCH), University Medical Center Hamburg-Eppendorf, Hamburg, Germany; 49 Department of Preventive Medicine, Seoul National University College of Medicine, Seoul, South Korea; 50 Department of Biomedical Sciences, Seoul National University College of Medicine, Seoul, South Korea; 51 Cancer Research Institute, Seoul National University, Seoul, South Korea; 52 Center for Medical Genetics, Ghent University, Ghent, Belgium; 53 Westmead Millenium Institute for Medical Research, University of Sydney, Sydney, Australia; 54 Sheffield Cancer Research, Department of Oncology, University of Sheffield, Sheffield, UK; 55 Academic Unit of Pathology, Department of Neuroscience, University of Sheffield, Sheffield, UK; 56 Department of Clinical Genetics, Fox Chase Cancer Center, Philadelphia, PA USA; 57 Department of Pathology, Leiden University Medical Center, Leiden, The Netherlands; 58 Department of Human Genetics, Leiden University Medical Center, Leiden, The Netherlands; 59 Oncogenetics Group, University Hospital Vall d’Hebron, Vall d’Hebron Institute of Oncology (VHIO) and Universitat Autònoma de Barcelona, Barcelona, Spain; 60 Department of Medicine, Abramson Cancer Center, Perelman School of Medicine, University of Pennsylvania, Philadelphia, PA USA; 61 Division of Cancer Epidemiology and Genetics, National Cancer Institute, Rockville, MD USA; 62 Department of Genetics, University of Pretoria, Pretoria, South Africa; 63 Clinics of Obstetrics and Gynaecology, Hannover Medical School, Hannover, Germany; 64 Department of Non-Communicable Disease Epidemiology, London School of Hygiene and Tropical Medicine, London, UK; 65 Genomics Center, Centre Hospitalier Universitaire de Québec Research Center, Laval University, Québec City, Canada; 66 Department of Biomedical Sciences, Faculty of Science and Technology, University of Westminster, London, UK; 67 Institute of Human Genetics, Muenster, Germany; 68 Division of Population Sciences, Moffitt Cancer Center & Research Institute, Tampa, FL USA; 69 Department of Oncology, Sahlgrenska University Hospital, Gothenburg, Sweden; 70 Department of Oncology, Rigshospitalet, Copenhagen University Hospital, Copenhagen, Denmark; 71 Genetic Medicine, Manchester Academic Health Sciences Centre, Central Manchester University Hospitals NHS Foundation Trust, Manchester, UK; 72 Department of Gynaecology and Obstetrics, University Hospital Erlangen, Friedrich-Alexander University Erlangen-Nuremberg, Comprehensive Cancer Center Erlangen-EMN, Erlangen, Germany; 73 David Geffen School of Medicine, Department of Medicine Division of Hematology and Oncology, University of California at Los Angeles, Los Angeles, CA USA; 74 Department of Breast Surgery, Herlev Hospital, Copenhagen University Hospital, Herlev, Denmark; 75 ANZ GOTG Coordinating Centre, Australia New Zealand GOG, Camperdown, NSW Australia; 76 Susanne Levy Gertner Oncogenetics Unit, Sheba Medical Center, Tel-Hashomer, Israel; 77 Section of Genetic Oncology, Deparment of Laboratory Medicine, University and University Hospital of Pisa, Pisa, Italy; 78 Department of Epidemiology, Shanghai Cancer Institute, Shanghai, China; 79 Cancer Risk and Prevention Clinic, Dana-Farber Cancer Institute, Boston, MA USA; 80 Division of Genetics and Epidemiology, The Institute of Cancer Research, London, UK; 81 Institute of Human Genetics, University Würzburg, Wurzburg, Germany; 82 INSERM U1052, CNRS UMR5286, Université Lyon, Centre de Recherche en Cancérologie de Lyon, Lyon, France; 83 Genetic Epidemiology of Cancer team, Inserm, U900, Institut Curie, Mines ParisTech, 75248 Paris, France; 84 Department of Tumour Biology, Institut Curie, Paris, France; 85 Institut Curie, INSERM U830, Paris, France; 86 Université Paris Descartes, Sorbonne Paris Cité, Paris, France; 87 GEMO study: National Cancer Genetics Network, UNICANCER Genetic Group, ᅟ, France; 88 Department of Pathology and Laboratory Medicine, University of Kansas Medical Center, Kansas City, KS USA; 89 Department of Dermatology, Huntsman Cancer Institute, University of Utah School of Medicine, Salt Lake City, UT USA; 90 Clinical Genetics Branch, Division of Cancer Epidemiology and Genetics, National Cancer Institute, National Institutes of Health, Rockville, MD USA; 91 Environmental Epidemiology of Cancer, Center for Research in Epidemiology and Population Health, INSERM, Villejuif, France; 92 University Paris-Sud, Villejuif, France; 93 Department of Preventive Medicine, Keck School of Medicine, University of Southern California, Los Angeles, CA USA; 94 Department of Health Sciences Research, Mayo Clinic, Rochester, MN USA; 95 Molecular Genetics of Breast Cancer, German Cancer Research Center (DKFZ), Heidelberg, Germany; 96 Center for Genomic Medicine, Rigshospitalet, Copenhagen University Hospital, Copenhagen, Denmark; 97 Cancer Center, Kuopio University Hospital, Kuopio, Finland; 98 Institute of Clinical Medicine, Pathology and Forensic Medicine, University of Eastern Finland, Kuopio, Finland; 99 Imaging Center, Department of Clinical Pathology, Kuopio University Hospital, Kuopio, Finland; 100 Saw Swee Hock School of Public Health, National University of Singapore, Singapore, Singapore; 101 Department of Surgery, National University Health System, Singapore, Singapore; 102 Cancer Research Initiatives Foundation, Subang Jaya, Selangor Malaysia; 103 Breast Cancer Research Unit, Cancer Research Institute, University Malaya Medical Centre, Kuala Lumpur, Malaysia; 104 Cancer Division, QIMR Berghofer Medical Research Institute, Brisbane, QLD Australia; 105 Family Cancer Clinic, Netherlands Cancer Institute, Amsterdam, The Netherlands; 106 The Hereditary Breast and Ovarian Cancer Research Group Netherlands (HEBON), Coordinating center: Netherlands Cancer Institute, Amsterdam, The Netherlands; 107 Suburban Hospital, Bethesda, MD USA; 108 Care of City of Hope Clinical Cancer Genetics Community Research Network, Duarte, CA USA; 109 Department of Medical Oncology, Family Cancer Clinic, Erasmus MC Cancer Institute, Rotterdam, The Netherlands; 110 Center for Medical Genetics, NorthShore University HealthSystem, Evanston, IL USA; 111 Program in Genetic Epidemiology and Statistical Genetics, Harvard School of Public Health, Boston, MA USA; 112 Department of Epidemiology, Harvard School of Public Health, Boston, MA USA; 113 N.N. Petrov Institute of Oncology, St. Petersburg, Russia; 114 Lombardi Comprehensive Cancer Center, Georgetown University, Washington, DC USA; 115 Division of Epidemiology and Prevention, Aichi Cancer Center Research Institute, Aichi, Japan; 116 Department of Genetics and Pathology, Pomeranian Medical University, Szczecin, Poland; 117 State Research Institute Centre for Innovative Medicine, Vilnius, Lithuania; 118 Department of Clinical Genetics, Aarhus University Hospital, Aarhus, N, Denmark; 119 Department of Epidemiology, Cancer Prevention Institute of California, Fremont, CA USA; 120 Department of Health Research and Policy - Epidemiology, Stanford University School of Medicine, Stanford, CA USA; 121 Stanford Cancer Institute, Stanford University School of Medicine, Stanford, CA USA; 122 Genomics Center, Centre Hospitalier Universitaire de Québec Research Center and Laval University, Quebec City, QC Canada; 123 Women’s Cancer Program at the Samuel Oschin Comprehensive Cancer Institute, Cedars-Sinai Medical Center, Los Angeles, CA USA; 124 Department of Pathology, Oulu University Hospital, University of Oulu, Oulu, Finland; 125 School of Medicine, National University of Ireland, Galway, Ireland; 126 Department of Obstetrics and Gynecology, Helsinki University Hospital, University of Helsinki, Helsinki, Finland; 127 Department of Genetics and Fundamental Medicine, Bashkir State University, Ufa, Russia; 128 Prosserman Centre for Health Research, Lunenfeld-Tanenbaum Research Institute of Mount Sinai Hospital, Toronto, Canada; 129 Division of Epidemiology, Dalla Lana School of Public Health, University of Toronto, Toronto, Canada; 130 Molecular Diagnostics Laboratory, IRRP, National Centre for Scientific Research “Demokritos”, Athens, Greece; 131 The Hong Kong Hereditary Breast Cancer Family Registry, Cancer Genetics Center, Hong Kong Sanatorium and Hospital, Hong Kong, China; 132 Department of Surgery, The University of Hong Kong, Hong Kong, China; 133 Vesalius Research Center, Leuven, Belgium; 134 Laboratory for Translational Genetics, Department of Oncology, University of Leuven, Leuven, Belgium; 135 Molecular Diagnostic Unit, Hereditary Cancer Program, IDIBELL (Bellvitge Biomedical Research Institute), Catalan Institute of Oncology, Barcelona, Spain; 136 University of Hawaii Cancer Center, Honolulu, HI USA; 137 Department of Surgery, Soonchunhyang University and Hospital, Seoul, South Korea; 138 Department of Molecular Medicine and Surgery, Karolinska Institutet, Stockholm, Sweden; 139 Division of Health Sciences, Warwick Medical School, Warwick University, Coventry, UK; 140 Unit of Medical Genetics, Department of Preventive and Predictive Medicine, Fondazione IRCCS (Istituto di Ricovero e Cura a Carattere Scientifico) Istituto Nazionale Tumori (INT), Milan, Italy; 141 Department of Oncology - Pathology, Karolinska Institutet, Stockholm, Sweden; 142 National Center for Tumor Diseases, University of Heidelberg, Heidelberg, Germany; 143 Department of Preventive Medicine, Kyushu University Faculty of Medical Sciences, Fukuoka, Japan; 144 Division of Gynaecology and Obstetrics, Technische Universität München, Munich, Germany; 145 Institute of Population Health, University of Manchester, Manchester, UK; 146 Laboratory Medicine Program, University Health Network, Toronto, ON Canada; 147 Department of Laboratory Medicine and Pathobiology, University of Toronto, Toronto, ON Canada; 148 Beckman Research Institute of City of Hope, Duarte, CA USA; 149 Carbone Cancer Center, University of Wisconsin, Madison, WI USA; 150 Cancer Prevention Program, Fred Hutchinson Cancer Research Center, Seattle, WA USA; 151 Department of Medicine and Genetics, University of California, San Francisco, San Francisco, CA USA; 152 Clinical Genetics Research Lab, Department of Cancer Biology and Genetics, Memorial Sloan-Kettering Cancer Center, New York, NY USA; 153 Department of Molecular Genetics, National Institute of Oncology, Budapest, Hungary; 154 Center for Clinical Cancer Genetics and Global Health, University of Chicago Medical Center, Chicago, IL USA; 155 Human Genetics Group, Human Cancer Genetics Program, Spanish National Cancer Centre (CNIO), Madrid, Spain; 156 Biomedical Network on Rare Diseases (CIBERER), Madrid, Spain; 157 Unit of Medical Genetics, Department of Biomedical, Experimental and Clinical Sciences, University of Florence, Florence, Italy; 158 Department of Immunology, Genetics and Pathology, Uppsala University, Uppsala, Sweden; 159 University Hospital Gashuisberg, Leuven, Belgium; 160 Unit of Medical Genetics, Department of Preventive and Predictive Medicine, Fondazione IRCCS (Istituto Di Ricovero e Cura a Carattere Scientifico) Istituto Nazionale Tumori (INT), Milan, Italy; 161 IFOM, Fondazione Istituto FIRC (Italian Foundation of Cancer Research) di Oncologia Molecolare, Milan, Italy; 162 Department of Obstetrics and Gynecology, and Comprehensive Cancer Center, Medical University of Vienna, Vienna, Austria; 163 Department of Cancer Epidemiology, Moffitt Cancer Center, Tampa, FL USA; 164 Unit of Molecular Bases of Genetic Risk and Genetic Testing, Department of Preventive and Predictive Medicine, Fondazione IRCCS (Istituto Di Ricovero e Cura a Carattere Scientifico) Istituto Nazionale Tumori (INT), Milan, Italy; 165 Section of Cancer Genetics, The Institute of Cancer Research, London, UK; 166 Department of Preventive Medicine, Keck School of Medicine, University of Southern California Norris Comprehensive Cancer Center, Los Angeles, CA USA; 167 Department of Basic Sciences, Shaukat Khanum Memorial Cancer Hospital and Research Centre (SKMCH & RC), Lahore, Pakistan; 168 Clalit National Israeli Cancer Control Center and Department of Community Medicine and Epidemiology, Carmel Medical Center and B. Rappaport Faculty of Medicine, Haifa, Israel; 169 Centre of Familial Breast and Ovarian Cancer, Department of Gynaecology and Obstetrics and Centre for Integrated Oncology (CIO), Center for Molecular Medicine Cologne (CMMC), University Hospital of Cologne, Cologne, Germany; 170 Obstetrics and Gynecology, Ohio State University College of Medicine, Columbus, OH USA; 171 National Cancer Institute, Bangkok, Thailand; 172 Research Oncology, Guy’s Hospital, King’s College London, London, UK; 173 Division of Molecular Gyneco-Oncology, Department of Gynaecology and Obstetrics, University Hospital of Cologne, Cologne, Germany; 174 Center of Familial Breast and Ovarian Cancer, University Hospital of Cologne, Cologne, Germany; 175 Center for Integrated Oncology, University Hospital of Cologne, Cologne, Germany; 176 Center for Molecular Medicine, University Hospital of Cologne, Cologne, Germany; 177 School of Public Health, China Medical University, Taichung, Taiwan; 178 Taiwan Biobank, Institute of Biomedical Sciences, Academia Sinica, Taipei, Taiwan; 179 Department of Obstetrics and Gynecology, Comprehensive Cancer Center, Medical University of Vienna, Vienna, Austria; 180 Centre Hospitalier Universitaire de Québec Research Center and Laval University, Quebec City, QC Canada; 181 Genetic Epidemiology Laboratory, Department of Pathology, University of Melbourne, Parkville, VIC Australia; 182 Hannover Medical School, Hannover, Germany; 183 Division of Breast Cancer Research, The Institute of Cancer Research, London, UK; 184 National Human Genome Research Institute, National Institutes of Health, Bethesda, MD USA; 185 Lunenfeld-Tanenbaum Research Institute of Mount Sinai Hospital, Toronto, ON Canada; 186 Department of Genetics, Portuguese Oncology Institute, Porto, Portugal; 187 Biomedical Sciences Institute (ICBAS), Porto University, Porto, Portugal; 188 Department of Epidemiology, Mailman School of Public Health, Columbia University, New York, NY USA; 189 Genetic Counseling Unit, Hereditary Cancer Program, IDIBELL (Bellvitge Biomedical Research Institute), Catalan Institute of Oncology, Barcelona, Spain; 190 Department of Clinical Genetics, Odense University Hospital, Odense, C, Denmark; 191 Latvian Biomedical Research and Study Centre, Riga, Latvia; 192 Program in Cancer Genetics, Departments of Human Genetics and Oncology, McGill University, Montreal, QC Canada; 193 Currently at Medical School Cambridge University, Cambridge, UK; 194 Department of Molecular Virology, Immunology and Medical Genetics, Comprehensive Cancer Center, The Ohio State University, Columbus, OH USA; 195 Department of Medical Oncology, Beth Israel Deaconess Medical Center, Boston, MA USA; 196 Department of Clinical Genetics, Erasmus University Medical Center, Rotterdam, The Netherlands; 197 Clinical Genetics Service, Department of Medicine, Memorial Sloan-Kettering Cancer Center, New York, NY USA; 198 Department of Obstetrics and Gynecology, University of Ulm, Ulm, Germany; 199 Clinical Cancer Genetics, for the City of Hope Clinical Cancer Genetics Community Research Network, Duarte, CA USA; 200 Laboratory of Cancer Genetics and Tumor Biology, Department of Clinical Chemistry and Biocenter Oulu, University of Oulu, Oulu, Finland; 201 Laboratory of Cancer Genetics and Tumor Biology, Northern Finland Laboratory Centre NordLab, Oulu, Finland; 202 Singapore Eye Research Institute, National University of Singapore, Singapore, Singapore; 203 Department of Medical Oncology, Papageorgiou Hospital, Aristotle University of Thessaloniki School of Medicine, Thessaloniki, Greece; 204 Department of Surgery, National Taiwan University Hospital, Taipei, Taiwan; 205 Department of Genetics, QIMR Berghofer Medical Research Institute, Brisbane, Australia; 206 Peter MacCallum Cancer Center, The University of Melbourne, Melbourne, Australia

**Keywords:** Fine-scale mapping, Genetic risk factor, *PTHLH*, *CCDC91*, Breast cancer, *BRAC1* mutation carriers

## Abstract

**Background:**

Multiple recent genome-wide association studies (GWAS) have identified a single nucleotide polymorphism (SNP), rs10771399, at 12p11 that is associated with breast cancer risk.

**Method:**

We performed a fine-scale mapping study of a 700 kb region including 441 genotyped and more than 1300 imputed genetic variants in 48,155 cases and 43,612 controls of European descent, 6269 cases and 6624 controls of East Asian descent and 1116 cases and 932 controls of African descent in the Breast Cancer Association Consortium (BCAC; http://bcac.ccge.medschl.cam.ac.uk/), and in 15,252 *BRCA1* mutation carriers in the Consortium of Investigators of Modifiers of *BRCA1/2* (CIMBA). Stepwise regression analyses were performed to identify independent association signals. Data from the Encyclopedia of DNA Elements project (ENCODE) and the Cancer Genome Atlas (TCGA) were used for functional annotation.

**Results:**

Analysis of data from European descendants found evidence for four independent association signals at 12p11, represented by rs7297051 (odds ratio (OR) = 1.09, 95 % confidence interval (CI) = 1.06–1.12; *P* = 3 × 10^-9^), rs805510 (OR = 1.08, 95 % CI = 1.04–1.12, *P* = 2 × 10^-5^), and rs1871152 (OR = 1.04, 95 % CI = 1.02–1.06; *P* = 2 × 10^-4^) identified in the general populations, and rs113824616 (*P* = 7 × 10^-5^) identified in the meta-analysis of BCAC ER-negative cases and *BRCA1* mutation carriers. SNPs rs7297051, rs805510 and rs113824616 were also associated with breast cancer risk at *P* < 0.05 in East Asians, but none of the associations were statistically significant in African descendants. Multiple candidate functional variants are located in putative enhancer sequences. Chromatin interaction data suggested that *PTHLH* was the likely target gene of these enhancers. Of the six variants with the strongest evidence of potential functionality, rs11049453 was statistically significantly associated with the expression of *PTHLH* and its nearby gene *CCDC91* at *P* < 0.05.

**Conclusion:**

This study identified four independent association signals at 12p11 and revealed potentially functional variants, providing additional insights into the underlying biological mechanism(s) for the association observed between variants at 12p11 and breast cancer risk.

**Electronic supplementary material:**

The online version of this article (doi:10.1186/s13058-016-0718-0) contains supplementary material, which is available to authorized users.

## Background

A previous genome-wide association study (GWAS) identified a common single nucleotide polymorphism (SNP), rs10771399 (termed the index SNP in this paper) at 12p11 to be associated with breast cancer risk in women of European descent [1]. This association, which did not vary by estrogen receptor (ER) status, was one of the most significant associations found for breast cancer risk in *Breast cancer 1* (*BRCA1*) mutation carriers so far, and the association was predominantly found in carriers with ER-negative (ER-(-)) breast cancer [2, 3]. This association was also replicated in East Asian women [4]. The index SNP lies in an approximately 300-kb linkage disequilibrium (LD) block, containing one known breast cancer associated gene that encodes parathyroid hormone-like hormone (*PTHLH*). This hormone has been shown to play a role in breast tumor initiation, progression, and metastasis in animal studies [5, 6] and was found to be associated with prognosis in breast cancer patients [7]. The index SNP, however, is located in a region with no evidence of functional significance [8]. The underlying biologic mechanisms and functional variants that drive the observed association have not yet been investigated. Furthermore, it is possible that additional independent risk signals may be present in the same region, as has been observed for other susceptibility regions [9–11]. In order to identify additional association signals at the12p11 locus with breast cancer risk, understand the underlying mechanisms and potential causal variants responsible for the association, we conducted a large fine-scale mapping study including data from 55,540 breast cancer cases and 51,168 controls in the Breast Cancer Association Consortium (BCAC) and 15,252 *BRCA1* mutation carriers in the Consortium of Investigators of Modifiers of *BRCA1/2* (CIMBA).

## Methods

### Study population

The BCAC included 40 studies of women of European descent (48,155 cases and 43,612 controls), nine of Asian descent (6269 cases and 6624 controls), and two of African-American descent (1116 cases and 932 controls). The CIMBA included 45 studies of women of European descent (15,252 *BRCA1* mutation carriers), of whom 7797 had been diagnosed with breast cancer. Details on the study characteristics, participant characteristics and the methodology used by the BCAC and CIMBA have been published elsewhere [12–14]. Ethical approval of each study was given by the local institutional review boards. The full names of the institutional review boards that approved each study were listed in the Additional file 1.

### SNP selection and genotyping

All SNPs within a 700-kb “fine mapping” interval at 12p11 (chr12: 27958733-28658733, hg19) were identified from the 1000 Genomes Project (1000G) (http://browser.1000genomes.org) CEU (April 2010) [15] and Hapmap III [16] (http://hapmap.ncbi.nlm.nih.gov/). The interval included all SNPs in LD (*r*^2^ > 0.1) with the target SNP rs197593 (*r*^2^ = 0.95 with the index SNP rs10771399) [1]. Tagging SNPs were selected to capture the remaining SNPs in the fine-mapping region at *r*^2^ > 0.9. After quality control, genotypes for 441 SNPs were available for analysis. To improve the coverage, imputation was performed using data from the 1000G (March 2012) as the reference and the program IMPUTE2 [17] (https://mathgen.stats.ox.ac.uk/impute/impute_v2.html). This was done separately for women of European, East Asian, and African descent and *BRCA1* mutation carriers. Using criteria of minor allele frequency (MAF) ≥2 % and an imputation quality *R*^2^ > 0.3, genotype data were generated for a total of 1634 SNPs for studies of European women, 1360 for studies of East Asian women, 2508 for studies of African women in BCAC and 1646 for studies of *BRCA1* mutation carriers in CIMBA.

### Statistical analysis

For BCAC studies, unconditional logistic regression models were used to estimate allelic odds ratios (OR) and their 95 % confidence intervals (CIs) of each of the SNPs included in the study. Analyses were performed separately for each ethnic group, and adjusted for study and principal components (seven for European studies and two each for Asian and African ancestry studies) [12]. Additional adjustment for age (age at diagnosis for cases and age at interview for controls) did not change the estimates, and thus age was not adjusted for in the main analyses. Tests of heterogeneity of the ORs across studies were conducted using Cochran’s *Q* test. To identify independent association signals, we performed forward stepwise selection analyses with all SNPs associated with breast cancer risk at *P* < 0.0001 in BCAC European descendants or at *P* < 0.005 for East Asian descendants in the single-marker analysis. To reduce type 2 errors, we used a less stringent statistical significance threshold because of the smaller sample size for East Asian descendants than for European descendants in this study. Pairwise SNP-SNP interactions were evaluated using the likelihood ratio test for all SNPs selected from the forward stepwise regression analysis. Stratified analyses by ER status were performed, and the heterogeneity was assessed by case-only analysis. We estimated haplotype frequencies using the haplo.stats package under R with the expectation-maximum (EM) algorithm [18] and estimated the haplotype-specific ORs for women of European descent with adjustment for studies and principal components as described above. To evaluate whether the association varied by early-onset and late-onset cancer, stratified analyses by age at cancer diagnosis (≥45 or <45 years) were performed. The familial relative risk (FRR, λ) associated with independently associated variants in this locus was calculated using the method described previously [19, 20].

For CIMBA studies, the associations between genetic variants and breast cancer risk were evaluated using a 1-degree of freedom (df) per allele trend test (*P*-trend), by modeling the retrospective likelihood of the observed genotypes conditional on breast cancer phenotypes [21]. To allow for the non-independence among related individuals, an adjusted test statistic was used, which took into account the correlation between study participants [22]. Per-allele hazard ratio (HR) estimates were obtained by maximizing the retrospective likelihood. All analyses were stratified by country of residence. To increase the statistical power to detect independent signals in *BRCA*1 mutation carriers, we conducted a meta-analysis of the BCAC and CIMBA studies [23]. Because approximately 80 % of breast tumors with known ER status in *BRCA1* mutation carriers were ER(-) [2], we only included the ER(-) breast cancer cases for BCAC studies. We combined the logarithm of the per-allele HR estimated in *BRCA1* mutation carriers and the logarithm of the per-allele OR estimated in BCAC using a fixed-effects model. We further determined whether there is evidence for independent association signals through a serial of conditional meta-analyses. We performed a conditional analysis on the top variant identified in the meta-analysis mentioned above in each consortium, and carried out the meta-analysis on the conditional *P* value for each variant to identify the most significant variant after conditioning on the top variant in the whole region. We continued to perform the conditional meta-analyses until the most significant association found had a *P* value >0.0001.

### Functional annotation

We used the Encyclopedia of DNA Elements (ENCODE) chromatin states (chromHMM) annotation, DNase I hypersensitive, transcription factor binding sites, histone modifications of epigenetic markers (H3K4Me1, H3K4Me3 and H3K27Ac) data from ENCODE [24] (http://genome.ucsc.edu/ENCODE/) to determine the likely regulatory elements. We used chromatin interaction analysis by paired end tag (ChIA-PET), genome conformation capture (Hi-C) data from ENCODE and enhancer-promoter interaction data predicted by He et al. [25] to identify putative gene targets in mammary cell lines (human mammary epithelial cells (HMEC) and Michigan Cancer Foundation-7 (MCF7)). We used maps of enhancers as defined in Corradin et al. [8] and Hnisz et al. [26] to identify the locations of potential enhancers. We obtained RNA-seq data from ENCODE, respectively, to evaluate the expression of protein-coding genes in mammary cell lines at this locus. We also used the same data in the chronic myeloid leukemia cell line (K562) as a comparison if available.

To predict the most likely functional variants, we mapped all candidates to the transcription factor binding maps generated by ENCODE [24], based on the hypothesis that causal variants alter the binding affinity of transcription factors. We prioritized variants that were located in binding sites of master transcription factors of breast cancer and disrupted binding motif of transcription factors. We also prioritized variants that were located in active promoter regions in mammary cell lines. Two publicly available tools, RegulomeDB [27] (see http://regulome.stanford.edu/) and HaploReg V3 [28] (see http://www.broadinstitute.org/mammals/haploreg/haploreg.php), were also used to evaluate those candidate functional variants.

### Expression quantitative trait loci (eQTL) analysis

The eQTL analyses in tumor tissues were performed as previously described [29, 30]. Briefly, we downloaded RNA-Seq V2, DNA methylation and SNP genotype data of 1006 breast cancer tumor tissues from The Cancer Genome Atlas (TCGA) data portal [26] (see http://cancergenome.nih.gov/). We log2-transformed the RNA-Seq by expectation-maximization (RSEM) value of each gene, and performed principal component adjustment of gene expression data to remove potential batch effects. Residual linear regression analysis was used to detect eQTLs while adjusting for methylation and copy number alterations (CNA), according to the approach proposed by Li et al. [29].

The eQTL analyses in 135 tumor-adjacent normal breast tissues were performed using data from the Molecular Taxonomy of Breast Cancer International Consortium (METABRIC) [31] as previously described [32]. Briefly, gene expression levels were measured by the Illumina HT12 v3 microarray platform. Genotyping was performed using the Affymetrix SNP 6.0 array. Imputation was performed using data from the 1000G (CEU, March 2012) as the reference. Linear regression was performed to evaluate the association between genotypes and gene expression levels using the R (http://www.r-project.org/) package Matrix eQTL [32].

## Results

### Association results among women of European ancestry

**Fig. 1:**
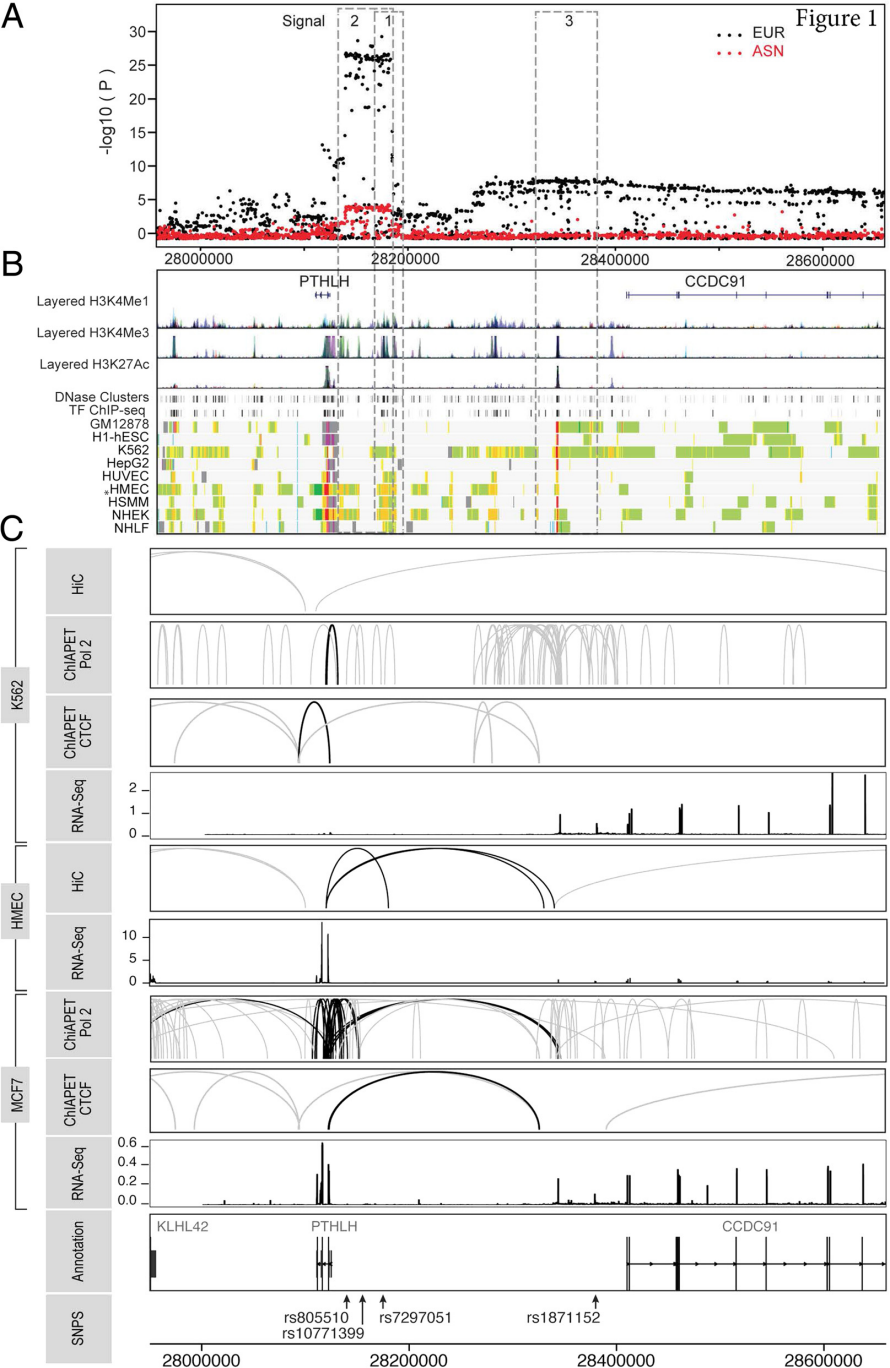
Genetic mapping and epigenetic landscape of the 12p11 locus (**a**). Regional association plot of the genotyped and imputed Illumina iSelect genotyping array of the Collaborative Oncological Gene-environment Study (iCOGS) genotype data. Three independent signals were identified, marked as signal 1, 2 and 3. **b** Functional annotations using data from the Encyclopedia of DNA Elements (ENCODE) project. From *top* to *bottom*, the epigenetic signals evaluated include histone modifications, DNase clusters, transcription factor ChIP-seq clusters, and ENCODE chromatin states (ChromHMM) in the ENCODE cell lines. The signals of different layered histone modifications from the same ENCODE cell line are shown in the same color (the detailed color scheme for each ENCODE cell line is described in the UCSC genome browser; http://genome.ucsc.edu). *Red* and *orange* in chromatin states represent active promoter and strong enhancer regions, respectively (the detailed color scheme of the chromatin states was described in the previous study [45]). All tracks were generated by the UCSC genome browser (hg 19). **c** Long-range chromatin interactions. From *top* to *bottom*, genome conformation capture (*Hi-C*), chromatin interaction analysis by paired end tag (*ChIA-PET*) and RNA-Seq data from K562 cell lines, Hi-C and RNA-Seq from human mammary epithelial cells (*HMEC*), ChIA-PET and RNA-Seq from MCF7 cell lines, gene annotations and single nucleotide polymorphism (*SNP*) annotations. *Black lines* represent interactions with the promoter region (-1500/+500) of *Parathyroid hormone-like hormone* (*PTHLH)*, and *gray lines* represent chromatin interaction that did not involve the *PTHLH* promoter region. The value of the RNA-Seq analysis corresponds to the mean reads per million (RPM) value for *PTHLH* from 65 K562, 4 HMEC and 19 MCF7 datasets, respectively. The annotation has been obtained through the Bioconductor annotation package TxDb.Hsapiens.UCSC.hg19.knownGene. The Hi-C and ChIA-PET raw data, available in the Gene Expression Omnibus (GEO) [GSE63525.K56, GSE33664, GSE39495], were processed using the GenomicRanges package. The tracks have been generated using ggplot2 and ggbio libraries in R

Of the 2075 SNPs evaluated, 833 were associated with breast cancer risk in women of European descent at *P* < 0.0001 (Fig. 1). Using forward stepwise selection, we identified two SNPs that were independently associated with breast cancer risk with conditional *P* < 0.0001, tagging two independent signals (Table 1, Fig. 1). The index SNP is located in signal 2, approximately 30 kb upstream of the *PTHLH* gene and was in strong LD with the lead SNP (rs805510) for this signal (*r*^2^ = 0.92). The lead SNP in signal 1, rs7297051, is located approximately 50 kb upstream of the *PTHLH* gene, and was in moderate LD with the index SNP (*r*^2^ = 0.42). The lead SNPs for signals 1 and 2 were moderately correlated (*r*^2^ = 0.36). After adjusting for the lead SNPs in signals 1 and 2, we found evidence of the presence of a third independent association signal (lead SNP rs1871152; conditional *P* = 2 × 10^-4^, Table 1, Fig. 1). Signal 3 lies approximately 60 kb upstream of another gene, *coiled-coil domain containing 91* (*CCDC91*). SNP rs1871152 was not correlated with the lead SNP in signal 1 or signal 2 (*r*^2^ = 0.01 for rs7297051 and *r*^2^ = 0.03 for rs805510). All lead SNPs for these three signals were associated with breast cancer risk at *P* < 5 × 10^-8^ in single-marker analyses (rs7297051 OR = 0.88, *P* = 4 × 10^-28^; rs805510 OR = 0.85, *P* = 10^-25^; rs1871152 OR = 0.94, *P* = 3 × 10^-8^). No apparent heterogeneity in the ORs of the identified SNPs across the 40 studies in BCAC was found (all *P*_heterogeneity_ > 0.75). No statistically significant interactions between any pair of these three lead SNPs were found (all *P* > 0.05).

**Table 1:** Independent association signals identified for breast cancer risk in the 12p11 locus in women of European ancestry

Signal	SNPs	Position (hg 19)	Alleles	EAF	LD (*r* ^2^)^b^	Univariate analysis		Conditional analysis		SNPs retained for functional annotation^e^
						Per-allele OR (95 % CI)^c^	*P*-trend	Per-allele OR (95 % CI)^d^	*P*-trend	
2	Index^a^ rs10771399	28155080	G*/A	0.12	-	0.85 (0.83–0.88)	5 × 10^-25^	-	-	-
1	rs7297051	28174817	T*/C	0.24	0.42	0.88 (0.86–0.90)	4 × 10^-28^	0.92 (0.89–0.94)	3 × 10^-9^	rs812020, chr12:28164044, rs2619434, rs2590275
2	rs805510	28139846	T*/C	0.12	0.88	0.85 (0.82–0.88)	10^-25^	0.93 (0.89–0.96)	2 × 10^-5^	74 SNPs^f^
3	rs1871152	28379826	G*/A	0.31	0.04	0.94 (0.92–0.96)	3 × 10^-8^	0.96 (0.94–0.98)	2 × 10^-4^	376 SNPs^g^

^*^Effect alleles. ^a^Identified in the initial genome-wide association study conducted in women of European descent [1]. ^b^Linkage disequilibrium (LD) with rs10771399 for women of European descent. ^c^Adjusted for studies, and the top principal components and an additional principal component accounting for the Leuven Multidisciplinary Breast Centre (LMBC) study. ^d^Included all three variants, and was adjusted for studies, and the top eight principal components as well as an additional principal component accounting for the LMBC study. ^e^Associated single nucleotide polymorphisms (SNPs) with a likelihood ratio >1/100 relative to the lead SNP in each signal. ^f^See Table S2 in Additional file 5. ^g^See Table S2 in Additional file 5. *EAF* effect allele frequency in controls, *OR* odds ratio, *CI* confidence interval

Using the lead SNP from each signal, rs805510, rs7297051 and rs1871152, we identified seven haplotypes with a frequency greater than 1 % (Table 2). The most common haplotype (frequency 51 %), carrying the major allele of each SNP, was used as the reference in the association analysis. The most statistically significant association was observed for the haplotype carrying the minor alleles at both signals 1 and 2 (TTA and TTG), while less pronounced yet significant associations were observed for individuals carrying the minor allele for signal 1 but not signal 2 (CTA and CTG), consistent with results for the independent association signals from the regression analyses. The evidence for signal 3 comes largely from the observation that the CCG haplotype, which carries the rare allele for signal 3 alone, was associated with reduced risk. The haplotype carrying only the minor allele in the lead SNP for signal 2 was too rare to evaluate. Stratified analyses revealed no evidence of any apparent heterogeneity in the association of these haplotypes with breast cancer risk by age at breast cancer diagnosis (age at diagnosis <45 vs ≥45 years).

**Table 2:** Associations between common haplotypes derived using lead single nucleotide polymorphisms and breast cancer risk in women of European ancestry

Haplotype	Overall breast cancer			Breast cancer (age at diagnosis <45 years)			Breast cancer (age at diagnosis ≥45 years)			*P*_heterogeneity_^b^
rs805510 - rs7297051- rs1871152	Frequency	OR (95 % CI)^a^	*P* value	Frequency	OR (95 % CI)^a^	*P* value	Frequency	OR (95 % CI)^a^	*P* value	
C-C-A	0.51	1.00 (Ref)	Ref	0.52	1.00 (Ref)	Ref	0.51	1.00 (Ref)	Ref	-
C-C-G	0.24	0.92 (0.89–0.95)	7 × 10^-8^	0.22	0.94 (0.89–1.00)	0.04	0.24	0.92 (0.89–0.95)	4 × 10^-7^	0.24
C-T-A	0.09	0.90 (0.87–0.95)	3 × 10^-6^	0.09	0.96 (0.89–1.03)	0.28	0.09	0.90 (0.86–0.94)	4 × 10^-7^	0.09
C-T-G	0.03	0.89 (0.82–0.96)	2 × 10^-3^	0.03	0.85 (0.73–0.98)	0.02	0.03	0.89 (0.82–0.96)	3 × 10^-3^	0.37
T-T-A	0.04	0.82 (0.77–0.88)	9 × 10^-9^	0.04	0.76 (0.67–0.87)	5 × 10^-5^	0.04	0.83 (0.76–0.85)	5 × 10^-8^	0.19
T-T-G	0.07	0.79 (0.76–0.83)	3 × 10^-23^	0.06	0.78 (0.71–0.85)	5 × 10^-8^	0.07	0.81 (0.77–0.85)	3 × 10^-18^	0.45
Rare	0.01	0.88 (0.79–0.99)	0.04	0.01	0.90 (0.72–1.13)	0.37	0.01	0.88 (0.78–0.99)	0.04	0.45

^a^Adjusted for studies and the top principal components. ^b^ *P* for heterogeneity between cases with age at diagnosis <45 years and ≥45 years. *Ref* reference

The associations of the three SNPs did not vary appreciably by ER status (Additional file 2: Table S3). In an attempt to identify potential independent association signals that might have been missed in the analysis described above that included all breast cancer cases (Table 1), we conducted forward stepwise regression analyses separately for ER(+) and ER(-) cases. For the ER(+) breast cancer, the lead SNPs for signals 1 and 2 were identical to those found for all cases combined. For signal 3, however, a different lead SNP (rs7959641) was identified, which was moderately correlated with rs1871152, the lead SNP identified in the overall analysis (*r*^2^ = 0.28) (Additional file 2: Table S3). The lead SNP for signal 3 in ER(-) cases is different from the SNP identified in all cases combined, but these two SNPs were highly correlated (*r*^2^ = 0.86) (Additional file 2: Table S3).

### Association results for *BRCA1* mutation carriers of European descent

Of the 2087 SNPs evaluated in the CIMBA among *BRCA1* mutation carriers of European descent, 234 were associated with breast cancer risk at *P* < 0.0001. The most significant association was found with rs113824616 (per-C allele HR 0.73, 95 % CI 0.64–0.82, *P* =1 × 10^-7^; Table 3). The three lead SNPs identified in BCAC had similar associations, although the association was statistically significant at *P* < 0.05 in conditional analyses only for the lead SNPs of signals 1 and 3 (rs7297051 and rs1871152, respectively) (Additional file 3: Table S4). Meta-analysis of data from BCAC ER(-) cases and CIMBA showed that rs113824616 was associated with breast cancer risk after adjusting for rs7297051 (conditional *P* = 7 × 10^-5^, *r*^2^ with rs10773199 = 0.40; Table 3). No additional independent signals were identified. We defined the association signal represented by SNP rs113824616 as signal 4.

**Table 3:** Independent association signals in the meta-analysis of BCAC (ER-) and *BRCA1* mutation carriers from CIMBA

	SNPs	Position (hg 19)	Alleles	EAF	LD (*r* ^2^)^§^	Univariate analysis		Conditional analysis	
						Per-allele effect (95 % CI)^a^	*P*-trend	Per-allele effect (95 % CI)^b^	*P*-trend
Index^ǂ^	rs10771399	28155080	G*/A	0.10	-	0.86 (0.80–0.91)	3 × 10^-6^	-	-
Meta-analysis of ER-negative cancer (BCAC + CIMBA)									
BCAC ER-									
Signal 1	rs7297051	28174817	T*/C	0.24	0.42	0.87 (0.83–0.91)	3 × 10^-10^	0.89 (0.85–0.94)	1 × 10^-5^
Signal 4	rs113824616	28184905	C*/T	0.05	0.40	0.75 (0.67–0.84)	5 × 10^-7^	0.86 (0.76–0.98)	0.02
CIMBA *BRCA1* mutation carriers									
Signal 1	rs7297051	28174817	T*/C	0.23	0.37	0.89 (0.85–0.93)	3 × 10^-7^	0.94 (0.90–0.98)	0.003
Signal 4	rs113824616	28184905	C*/T	0.04	0.49	0.73 (0.64–0.82)	1 × 10^-7^	0.83 (0.74–0.93)	0.001

Effect for Breast Cancer Association Consortium (BCAC): odds ratio; effect for Consortium of Investigators of Modifiers of *BRCA1/2* (CIMBA) cohort: hazard ratio. ^*^Effect alleles. ^a^Adjusted for studies, and the top principal components. ^b^Included both variants, and adjusted for studies and the top principal components. *SNPs* single nucleotide polymorphisms, *EAF* effect allele frequency in the or (BCAC) controls, *LD* linkage disequilibrium, *CI* confidence interval, *ER* estrogen receptor. ^§^represents LD with the index SNP rs10771399. ^ǂ^represented the index SNP, Identified in the initial genome-wide association study conducted in women of European descent [1]

### Association results among women of East Asian ancestry

Of the 1801 SNPs evaluated, 118 were associated with breast cancer risk in women of East Asian ancestry (*P* < 0.005) (Fig. 1). The four lead SNPs in European descendants had a similar association with breast cancer risk in East Asian women, although the association was statistically significant at *P* < 0.005 only for the lead SNPs of signals 1 and 2 (rs7297051 and rs805510, respectively) (Additional file 4: Table S5). The MAFs for the lead SNPs of signals 1, 2 and 4 were similar to those in Europeans, but the MAF for signal 3 (rs1871152) was markedly lower in East Asians. In conditional regression analyses, only the association with signal 1 was independently statistically significant, perhaps due to the small sample size. The per-allele ORs did not differ materially from those in Europeans in the conditional analysis (data not shown).

The most significant association in Asians was with SNP rs2737455 (MAF = 0.17, per-major (T) allele OR = 1.16, 95 % CI 1.09–1.25, *P* = 10^-5^). Among women of East Asian descent, this SNP was in high LD with the two lead SNPs for signals 1 and 2 identified in populations of European ancestry, rs7297051 (*r*^2^ = 0.67) and rs805510 (*r*^2^ = 0.84). This variant was also associated with breast cancer in women of European descent (per T-allele OR = 1.17, 95 % CI 1.14–1.21, *P* = 5 × 10^-25^). No additional independent signal was found on stepwise regression.

### Association results for women of African ancestry

Of the 2949 SNPs evaluated in African descendants, 116 were statistically significantly associated with breast cancer risk at *P* < 0.05. The most significant association was with rs10843021 (MAF = 0.38, per-C allele OR = 1.22, 95 % CI 1.08–1.39, *P* = 0.001), which is located 60 kb downstream of the gene *PTHLH.* This SNP is not in LD with any of the lead SNPs identified for women of European or East Asian descent (all *r*^2^ < 0.02). There was some evidence of association of this SNP with breast cancer risk in women of European descent (*P* = 8 × 10^-5^) but not in women of Asian descent (*P* = 0.23). None of the lead SNPs identified for women of European or East Asian descent were associated with breast cancer risk at *P <* 0.05 in African descendants, although the directions of the associations were consistent and the effect sizes did not differ significantly (Additional file 4: Table S5). The MAF of the index SNP rs10771399 (MAF = 0.04) was much lower in African descendants than that in Asian and European descendants (*P* < 0.001).

### Functional annotation

To identify putative causal variants, we used data from European descendants to exclude any variants that had a likelihood ratio <1/100 relative to the most significantly associated SNP in each signal (33). Based on this threshold, four variants in signal 1, 74 variants in signal 2, 376 variants in signal 3, and 2 variants in signal 4 were retained as candidates for causal variants (Fig. 1a and Additional file 5: Table S2).

Using data from ENCODE, we found that the histone markers (H3K27Ac and H3K4Me3) were enriched in each signal (Fig. 1b). Using both ChIA-PET chromatin interaction data and Hi-C data from ENCODE, we identified multiple and dense chromosomal interactions of variants at signals 1 and 2 with the promoter region of *PTHLH* in MCF7 cells (Fig. 1c). There was some evidence of interaction of variants at signal 3 with the promoter of *PTHLH* (Fig. 1c).

**Fig. 2:**
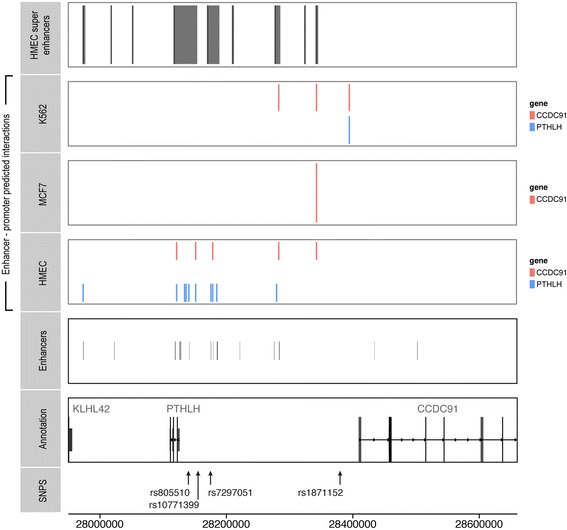
Enhancer-promoter interaction data at 12p11. From *top* to *bottom*, enhancer locations as defined by Corradin et al. [8] and Hnisz et al. [26] are shown in human mammary epithelial cells (*HMEC*) cell lines. Enhancer-promoter (EP)-predicted interactions as defined by He et al. [25] are shown in K562, MCF7 and HMEC cells. Gene annotations and single nucleotide polymorphism (*SNP*) annotations. *Orange* EP interactions are those with the *coiled-coil domain containing 91* (*CCDC91*) gene; *blue* EP are those with *Parathyroid hormone-like hormone* (*PTHLH*)

Using maps of predicted enhancer regions produced by Hnisz et al. [26] and Corradin et al. [8], we found that multiple candidate variants were located in enhancer regions in mammary cell lines (Fig. 2). Using predicted enhancer-promoter interaction data in HMEC and MCF7 cell lines generated by He et al. [25] (Fig. 2), we identified two interacting genes of these enhancers, *CCDC91* and *PTHLH*.

**Fig. 3:**
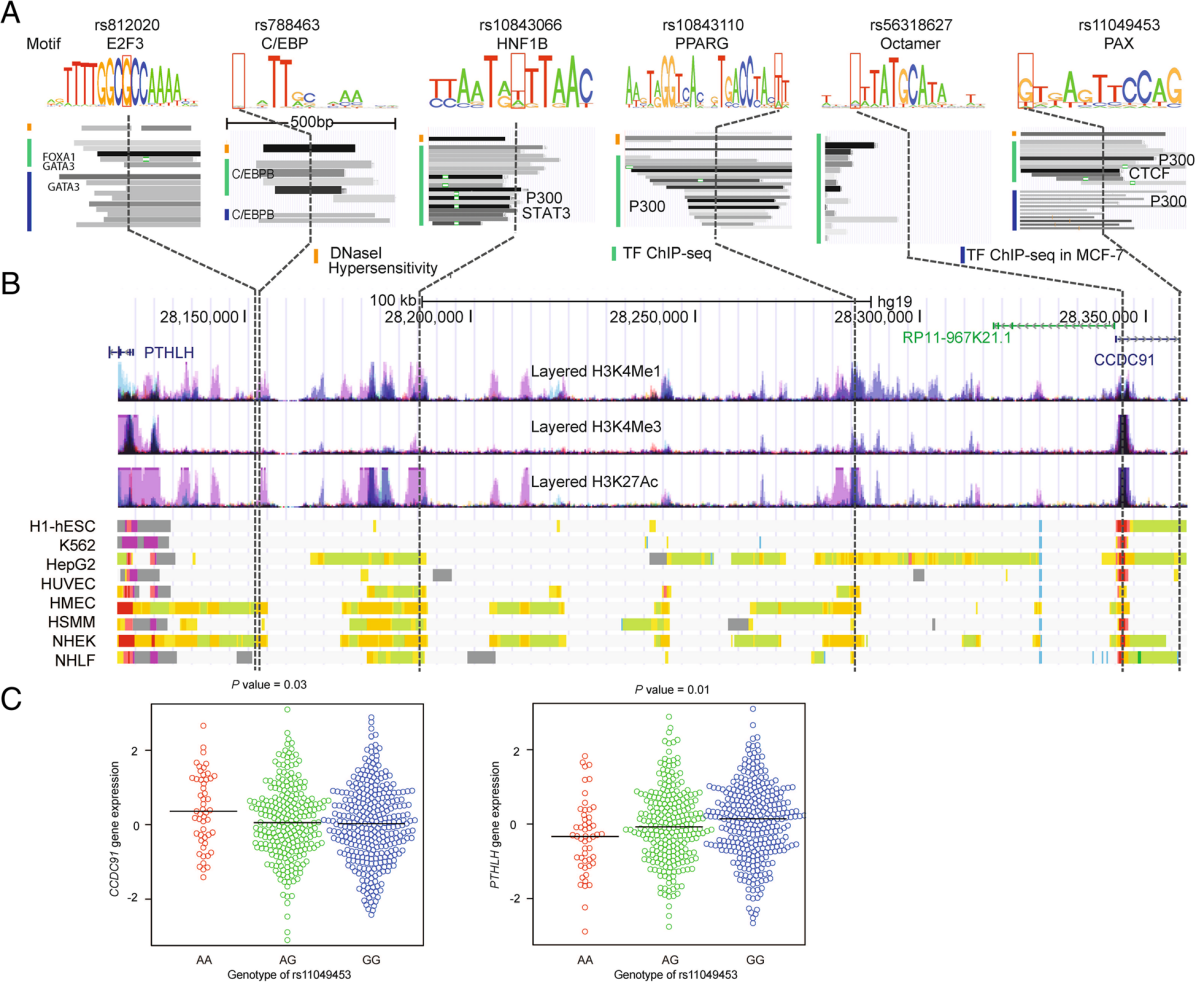
Putative functional variants and association of rs11049453 with gene expression in breast tumor tissues. **a** Epigenetic signals of five potential functional variants. From *top* to *bottom*, lanes showing that those variants mapped to transcription factors predicted binding motifs, DNase I hypersensitivity sites and transcription factor ChIP-Seq binding peaks in the Encyclopedia of DNA Elements (ENCODE) cell lines and MCF7. The corresponding location of each variant is indicated by a dashed line. **b** Epigenetic landscape at the 12p11 locus for breast cancer risk. From *top* to *bottom*, RefSeq genes (*PTHLH* and *CCDC91*), layered H3K4Me1, H3K4Me3 and H3K27Ac histone modifications and annotation using chromatin states on the ENCODE cell lines. The signals of different layered histone modifications from the same ENCODE cell line are shown in the same color (the detailed color scheme for each ENCODE cell line is described in the UCSC genome browser). *Red* and *orange* in the chromatin states represent the active promoter and strong enhancer regions, respectively (the detailed color scheme of the chromatin states was described in the previous study [45]). **c** rs11049453 and the expression of *coiled-coil domain containing 91* (*CCDC91*) and *parathyroid hormone-like hormone* (*PTHLH*). The association of the genotypes and the expression level of each gene was evaluated by residual linear regression [29]. *bp* base pairs, *C/EBP* CCAAT/enhancer-binding protein, *E2F3* E2F transcription factor 3, *HNF1B* HNF1 homeobox B, *PPARG* peroxisome proliferator-activated receptor gamma, *PAX* paired box

We next overlaid these candidate variants to the transcription factor binding site maps generated from ENCODE. We identified rs812020 within signal 1, rs788463 and rs10843066 within signal 2, and rs10843110, rs56318627 and rs11049453 within signal 3 to be the most likely functional variants (Fig. 3a and b; Additional file 6: Table S6). These SNPs were within or close to binding sites of multiple breast cancer-related transcription factors. Furthermore, these SNPs were predicted to disrupt the binding motifs recognized by transcription factors (Fig. 3a and b), suggesting a regulatory role. For example, in signal 1, rs812020 (per C-allele OR = 0.89, 95 % CI 0.87–0.91, *P* = 2 × 10^-27^) was annotated to a region bound by multiple key transcription factors for breast cancer, including GATA3 and FOXA1 (Fig. 3a and b). This SNP is predicted to disrupt the binding motif recognized by the transcription factor E2F3 and may change its binding affinity [32]. E2F3 has been found to increase centrosome amplification in mammary epithelial cells and regulate breast tumor development and metastasis [33]. In signal 3, SNP rs11049453 (per G-allele OR = 1.06, 95 % CI 1.04–1.08, *P* = 9 × 10^-8^) was in the binding site of transcription factors P300 and CTCF in MCF7 cell lines [31] (Fig. 3). It was also predicted to disrupt the binding motif of paired box (PAX) [33], which has been associated with the progression of breast cancer [34, 35]. No functional significance of the candidate variants in signal 4 was found.

To further explore the potential target genes, we performed eQTL analysis in both breast tumor and normal tissues. Using data on tumor tissues from TCGA, we found that rs10843110, rs56318627 and rs11049453 within signal 3 were associated with the expression of *PTHLH* at *P* < 0.05 and *CCDC91* at *P* < 0.10 (Additional file 7: Table S7). Among these highly correlated SNPs, the most significant association was found for rs11049453: the risk allele G of rs11049453 was associated with increased expression of *PTHLH* (*P* = 0.01) and decreased expression of *CCDC91* (*P* = 0.03, Fig. 3c). However, we did not find any statistically significant association for these six variants using data from adjacent normal breast tissues from METABRIC (all *P* > 0.05).

## Discussion

Through a fine-scale mapping study at 12p11, we identified four independent association signals for breast cancer risk in women of European descent. It is of interest that the fourth signal was identified only through the meta-analysis of ER(-) breast cancer and *BRCA1* mutation carriers, suggesting that this signal may be more specific to ER(-) cancers. The associations of these signals were in general consistent in women of European and East Asian descent.

Multiple genetic studies have confirmed that a locus at 12p11 is associated with breast cancer risk [2, 4]. However, it remained unknown whether the observed association was due to a single or multiple causal variants at this locus. In this study, we demonstrated that there were at least four independent signals at 12p11, three 100 kb upstream of the gene *PTHLH* (signals 1, 2 and 4), and one 60 kbp upstream from the gene *CCDC91* (signal 3), suggesting that there may be multiple causal variants and multiple underlying mechanisms for the observed association at the 12p11 locus. Furthermore, we identified multiple candidate causal variants at each signal: four in signal 1, 74 in signal 2, 376 in signal 3 and 2 in signal 4. Using functional genomic data from ENCODE, we observed that multiple candidate functional variants located in enhancer regions, and identified *PTHLH* and *CCDC91* as the likely target genes for these enhancers. Using data on transcription factor binding, we identified six putative functional variants with strong evidence of regulation of gene expression. Among these six variants, we observed that the rs11049453 was significantly associated with the expression of *PTHLH* and *CCDC91*. However, we could not exclude the possibility that there were other functional variants and other target genes at this locus.

*PTHLH* encodes the protein PTHrP, which has intracrine, autocrine or paracrine action in most normal tissues; its downstream effects include promotion of growth and anti-apoptotic effects [36]. It is a cause of humoral hypercalcemia of malignancy [37], and is expressed in more than two thirds of breast tumor tissue samples [7, 38]. It has been shown to affect the regulation of tumor-related genes, and is thought to affect the proliferation and migration of breast cancer cells [39]. PTHrP plays an important role in the formation of osteolytic bone metastases in breast cancer through its action on osteoblasts to increase RANK-ligand and promote osteoclast formation [40]. It has been proposed that PTHrP may promote breast cancer tumorigenesis; however, previous studies had conflicting results [41]. Less is known about the function of the *CCDC91* gene, which is located approximately 232 kb from the *PTHLH* gene. *CCDC91* encodes a protein known as p56 accessory protein or GGA binding partner, which binds proteins, and facilitates the transportation of secreted proteins through the trans-Golgi network [42]. *CCDC91* is also expressed in a variety of cancer cell lines including MCF7 [43]. Using cBioPortal (http://www.cbioportal.org/public-portal/), we found that both *PTHLH* and *CCDC91* genes were altered in breast tumors and that there was a statistically significant co-occurrence of alternations (including mutations and copy number aberrations) in both genes (*P* for tendency towards co-occurrence <0.001). Together with our findings, these results suggest that there might be correlation between these two genes and that alterations in both genes might contribute concurrently to breast cancer susceptibility. Future studies evaluating both genes and their interrelationship are needed to elucidate the underlying mechanism.

Functional annotation data suggested that the functional variants underlying the observed association, mainly those in signal 2, are located in enhancer regions involved in the transcriptional regulation of *PTHLH* and *CCDC91* in the MCF7 and HMEC cells. Moreover, we did not find similar functional evidence for the same region in the K562 cells, which suggests that the regulatory effects might be context-specific. We identified multiple putative functional variants associated with transcriptional factors that have been found to be important for breast cancer, including GATA3, FOXA1, C/EBP, P300 and STAT3, and overlapped with binding motifs of transcriptional factors, including E2F3, C/EBP, HNF1B, PPARG and PAX. Despite strong evidence for altering the binding of transcription factor and regulating gene transcription, we found only one eQTL among these putative functional variants, which lies in signal 3, suggesting that the underlying functional variants might exert a more subtle regulatory effect on gene expressions than expected. Although we found strong genetic and epigenetic evidence for potential functional variants in signals 1 and 2, we did not observe statistically significant association between these variants and the expression of *PTHLH* or *CCDC91*, or any other protein-coding genes within a flanking region of 500 kb for each variant. It is possible that the causal variants in these two signals might be involved in regulating non-coding genes or more distant genes. Future functional studies that comprehensively investigate the regulatory elements at these loci and their target genes will be needed to elucidate the molecular mechanisms.

The top risk variants identified in women of Asian and European ancestry were not associated with breast cancer risk in African descendants. It is possible that these top risk variants might not be correlated with the causal variants in African descendants due to their different LD structures. For example, the effect allele frequencies (EAFs) for the index SNP rs10773199 and the top risk variant rs805510 in African descendants were 0.04 and 0.45, respectively, and the EAFs for these two SNPs were similar in European descendants (EAF = 0.12 for both SNPs) and in East Asian descendants (EAF = 0.17 and 0.15, respectively), suggesting a distinct LD structure at this locus in African descendants. Similarly, the EAF for the SNP rs113824616 in African descendants (EAF = 0.01) was substantially lower than that in European descendants (EAF = 0.05). In addition, the sample size for African descendants included in this study was small and the power to detect the association of these variants was low. A previous fine-mapping study in African Americans with a larger sample size (3016 cases/2745 controls) than our study (1116 cases/932 controls) showed that rs10773199 is marginally associated with breast cancer risk (OR = 0.84, *P* = 0.089) [44], suggesting that there might be an association of the 12p11 locus with breast cancer risk in African descendants. Studies with a large sample size are needed to elucidate the association between this locus and breast cancer risk in African descendants.

To date this is the largest and most comprehensive fine-mapping study of the 12p11 region in relation to breast cancer risk. By using densely genotyped data from a very large number of cases and controls of European descent, we derived highly reliable estimates of the association between each common SNP and breast cancer risk in women of European descent. The sample size was relatively small for East Asian and African descendants, and associations with risk of overall breast cancer and molecular subtypes in these populations should be further evaluated in future larger studies.

## Conclusions

Through fine-mapping of the 12p11 locus, we identified multiple independent association signals for breast cancer risk. We estimate that the four independent signals identified by this study explain approximately 1 % of the familial relative risk of breast cancer in populations of European ancestry, more than doubling the risk explained by the index SNP (0.4 %). Bioinformatics analyses revealed that these signals are mapped to enhancer regions that interact with the gene *PTHLH and CCDC91.* We identified putative functional variants that might contribute to the observed association. Our findings also suggest a possible interrelation between *PTHLH* and *CCDC91* in the etiology of breast cancer. Our study has expanded the knowledge of genetic risk associated with breast cancer at the 12p11 locus and provided clues for future functional characterization.

## Abbreviations

BCAC, Breast Cancer Association Consortium; *BRCA1*, Breast cancer 1; C/EBP, CCAAT/enhancer-binding protein; *CCDC91*, Coiled-coil domain containing 91; ChIA-PET, chromatin interaction analysis by paired end tag; CI, confidence interval; CIMBA, Consortium of Investigators of Modifiers of *BRCA1/2*; CNA, copy number alterations; E2F3, E2F transcription factor 3; EAF, effect allele frequency; EM, expectation-maximum; ENCODE, Encyclopedia of DNA Elements; eQTL, expression quantitative trait loci; ER, estrogen receptor; FOXA1, forkhead box A1; GATA3, trans-acting T-cell-specific transcription factor GATA-3; GWAS, genome-wide association study; Hi-C, genome conformation capture; HMEC, human mammary epithelial cells; HNF1B, HNF1 homeobox B; HR, hazard ratio; iCOGS, Illumina iSelect genotyping array of the Collaborative Oncological Gene-environment Study; IMPUTEv2, IMPUTE version 2; LD, linkage disequilibrium; MAF, minor allele frequency; MCF7, Michigan Cancer Foundation-7; METABRIC, Molecular Taxonomy of Breast Cancer International Consortium; OR, odds ratio; PAX, paired box; PPARG, peroxisome proliferator-activated receptor gamma; PTHLH, parathyroid hormone-like hormone; QC, quality control; SNP, single nucleotide polymorphism; STAT3, signal transducer and activator of transcription 3; TCGA, The Cancer Genome Atlas

## Additional files

Additional file 1: Table S1. Ethical committees that approved each study. (PDF 94 kb) Additional file 2: Table S3. Independent association signals for risk of estrogen (ER)-positive and ER-negative breast cancer in European descendants. (PDF 47 kb) Additional file 3: Table S4. Associations of independent signals for breast cancer risk for BRCA1 mutation carriers. (PDF 64 kb) Additional file 4: Table S5. Associations of independent signals for breast cancer risk in women of East Asian and African descent. (PDF 66 kb) Additional file 5: Table S2. List of the variants that were retained for further functional annotation in European descendants. (PDF 54 kb) Additional file 6: Table S6. Putative functional SNPs identified using the ENCODE data. (PDF 50 kb) Additional file 7: Table S7. Gene expression analysis for putative functional SNPs using 1,006 breast tumor samples in TCGA. (PDF 46 kb)
