# Supplementary material for: Identification of independent association signals and putative functional variants for breast cancer risk through fine-scale mapping of the 12p11 locus
Source: Breast Cancer Res. 2016 Jun 21;18:64. doi: 10.1186/s13058-016-0718-0 (PMC4962376; doi:10.1186/s13058-016-0718-0)
Supplement: Additional file 1: Table S1. — Ethical committees that approved each study. (PDF 94 kb) [file 13058_2016_718_MOESM1_ESM.pdf]

Table S1 Ethical committees that approved each study

Study	Country	Approval Committee
Australian Breast Cancer Family Study (ABCFS)	Australia	The University of Melbourne Health Sciences Human Ethics Sub-Committee (HESC)
Amsterdam Breast Cancer Study (ABCS)	Netherlands	Leiden University Medical Center (LUMC) Commissie Medische Ethiek and Protocol Toetsingscommissie van het Nederlands Kanker Instituut/Antoni van Leeuwenhoek Ziekenhuis
Australian Breast Cancer Tissue Bank (ABCTB)	Australia	The Royal Prince Alfred Hospital Zone Ethics Committee in New South Wales, Australia
Asia Cancer Program (ACP)	Thailand	Ethics Committee of National Cancer Institute Thailand; Prince of Songkla University Faculty of Medicine Ethics Committee; Khon Kaen University Ethics Committee for Human Research; HRH Princess Maha Chakri Sirindhorn Medical Centre (MSMC) Ethics Committee
Bavarian Breast Cancer Cases and Controls (BBCC)	Germany	Friedrich-Alexander-Universitat Erlangen-Nurnberg Medizinische Fakultat Ethik-Commission
British Breast Cancer Study (BBCS)	UK	South East Multi-Centre Research Ethics Committee
Breast Cancer In Galway Genetic Study (BIGGS)	Ireland	Galway University College Hospital Clinical Research Ethical Committee
Breast Cancer Study of the University Clinic Heidelberg (BSUCH)	Germany	Medizinische Fakultat Heidelberg Ethikkommission
CECILE Breast Cancer Study (CECILE)	France	Comite Consultatif de Protection des Personnes dans la Recherche Biomedicale de Bicetre
Copenhagen General Population Study (CGPS)	Denmark	Kobenhavns Amt den Videnskabsetiske Komite
Spanish National Cancer Centre Breast Cancer Study (CNIO-BCS)	Spain	Hospital Universitario La Paz Comite Etico de Investigacion Clinica
California Teachers Study (CTS)	USA	UC Irvine: Office of Research Institutional Review Board
DEMOKRITOS	Greece	National Centre of Scientific Research "Demokritos" Ethics Committee and Aristotle University of Thessaloniki Medical School Ethics Committee
ESTHER Breast Cancer Study (ESTHER)	Germany	Ruprecht-Karls-Universitat Medizinische Fakultat Heidelberg Ethikkommission
German Consortium for Hereditary Breast & Ovarian Cancer (GC-HBOC)	Germany	Ethik-Kommission der Medizinischen Fakultat der Universitat zu Koln
Gene Environment Interaction and Breast Cancer in Germany (GENICA)	Germany	Rheinische Friedrich-Wilhelms-Universitat Medizinische Einrichtungen Ethik-Kommission
Helsinki Breast Cancer Study (HEBCS)	Finland	Helsingin ja uudenmaan sairaanhoitopiiri (Helsinki University Central Hospital Ethics Committee)
Hospital-based Epidemiologic Research Program at Aichi Cancer Center (HERPACC)	Japan	Ethics Committee for Human Genome Study at Aichi Cancer Center
Hannover-Minsk Breast Cancer Study (HMBCS)	Belarus	Medizinische Hochschule Hannover Ethik-Kommission
Karolinska Breast Cancer Study (KARBAC)	Sweden	Lokala Forskningsetikkommitten Nord
Kuopio Breast Cancer Project (KBCP)	Finland	Pohjois-Savon Sairraanhoitopiirin Kuntayhtyma Tutkimuseettinen Toimikunta
Kathleen Cuningham Foundation Consortium for Familial Breast Cancer/Australian Ovarian Cancer Study	Australia	kConFab: The Queenland Institute of Medical Research Human Research Ethics Committee (QIMR-HREC)

(kConFab/AOCS)		AOCS: Peter MacCallum Cancer Centre Ethics Committee
Los Angeles County Asian-American Breast Cancer Case-Control Study (LAABC)	USA	University of Southern California Health Sciences Campus IRB
Leuven Multidisciplinary Breast Centre (LMBC)	Belgium	Commissie Medische Ethiek van de Universitaire Ziekenhuizen Kuleuven
Mammary Carcinoma Risk Factor Investigation (MARIE)	Germany	Ruprecht-Karls-Universitat Medizinische Fakultat Heidelberg Ethikkommission
Milan Breast Cancer Study Group (MBCSG)	Italy	Comitato Etico Indipendente della Fondazione IRCCS "Istituto Nazionale dei Tumori"
Mayo Clinic Breast Cancer Study (MCBCS)	USA	Mayo Clinic IRB
Melbourne Collaborative Cohort Study (MCCS)	Australia	The Cancer Council Victoria Human Research Ethics Committee
Multi-ethnic Cohort (MEC)	USA	University of Southern California Health Sciences Campus IRB
Montreal Gene-Environment Breast Cancer Study (MTLGEBCS)	Canada	McGill University IRB, CSSS Dorval-Lachine-LaSalle Research Ethics Committee, CHU Sainte-Justine Research Ethics Committee, Centre Hospitalier de l'Universite de Montreal Research Ethics Committee, CSSS Sud-Ouest-Verdun Research Ethics Committee, CSSS d'Ahuntsic et Montreal-Nord Research Ethics Committee, CSSS de l'Ouest-de-l'Ile Research Ethics Committee, Centre Hospitalier Universitaire de Quebec Research Ethics Committee, Hopital Maisonneuve-Rosemont Research Ethics Committee, Hopital Santa Cabrini Research Ethics Committee, CSSS Coeur-de-l'Ile Research Ethics Committee.
Malaysian Breast Cancer Genetic Study (MYBRCA)	Malaysia	University Malaya Medical Centre Medical Ethics Committee
Norwegian Breast Cancer Study (NBCS)	Norway	Regional Komite for Medisinsk Forskningsetikk (Helseregion III Universitetet I Bergen, Universitetet I Oslo, Helseregion Sor, Helseregion II, and Ost-Norge)
Nashville Breast Health Study (NBHS)	USA	Vanderbilt University IRB
Oulu Breast Cancer Study (OBCS)	Finland	Ethical Committee of the Medical Faculty of University of Oulu and Northern Ostrobothnia Hospital District Ethical Committee
Ontario Familial Breast Cancer Registry (OFBCR)	Canada	Mount Sinai Hospital Research Ethics Board
Leiden University Medical Centre Breast Cancer Study (ORIGO)	Netherlands	Medical Ethical Committee and Board of Directors of the Leiden University Medical Center (LUMC)
The Stefanie Spielman Breast Bank and the Columbus Area Control Sample Bank, Ohio State University (OSU)	USA	OSU Cancer Institutional Review Board
NCI Polish Breast Cancer Study (PBCS)	Poland	National Institute of Health (NIH) IRB
Karolinska Mammography Project for Risk Prediction of Breast Cancer-prevalent cases (pKARMA)	Sweden	Regionala Etikprovningsnamnden i Stockholm (Regional Ethical Review Board in Stockholm)
Rotterdam Breast Cancer Study (RBCS)	Netherlands	Medische Ethische Toetsings Commissie Erasmus Medisch Centrum
Roswell Park Cancer Center biorepository, Roswell Park Cancer Institute (RPCI)	USA	RPCI Institutional Review Board
Singapore and Sweden Breast Cancer Study (SASBAC)	Sweden	Regionala Etikprovningsnamnden i Stockholm (Regional Ethical Review Board in Stockholm)
Shanghai Breast Cancer Genetic Study (SBCGS)	China	Shanghai Cancer Institute, Shanghai Center for Disease Prevention and Control IRB and Vanderbilt University Medical Center IRB
Sheffield Breast Cancer Study (SBCS)	UK	South Sheffield Research Ethics Committee
Southern Community Cohort Study (SCCS)	USA	Vanderbilt University IRB and Meharry Medical College IRB

Study of Epidemiology and Risk factors in Cancer Heredity (SEARCH)	UK	Multi Centre Research Ethics Committee (MREC)
Seoul Breast Cancer Study (SEBCS)	Korea	Seoul National University College of Medicine/Seoul National University Hospital IRB
Singapore Breast Cancer Cohort (SGBCC)	Singapore	National Health Group (NHG) Domain Specific Review Board (DSRB)
Städtisches Klinikum Karlsruhe Deutsches Krebsforschungszentrum Study (SKKDKFZS)	Germany	Ethics Commission of the Medical Faculty of Heidelberg
IHCC-Szczecin Breast Cancer Study (SZBCS)	Poland	Komisji Bioetycznej Pomorskiej Akademii Medycznej
IARC-Thai Breast Cancer Study (TBCS)	Thailand	IARC Institutional Review Board Committee
Taiwanese Breast Cancer Study (TWBCS)	Taiwan	Human Subject Research Ethics Committee/IRB Academia Sinica
UK Breakthrough Generations Study (UKBGS)	UK	South East Multi-Centre Research Ethics Committee
Breast Cancer Family Registry (BCFR)	USA	Institutional Review Board University of Utah
(BCFR - addtional)	Australia	The University of Melbourne Health Sciences Human Ethics Sub-Committee
(BCFR - addtional)	USA	Columbia University Medical Center Institutional Review Board
(BCFR - addtional)	USA	Northern Californa Cancer Center Institutional Review Board
(BCFR - addtional)	Canada	University Health Network Research Ethics Board
(BCFR - addtional)	Canada	Mount Sinai Hospital Research Ethics Board
Baltic Familial Breast and Ovarian Cancer Consortium (BFBOCC)	Latvia, Lithuania	Centrālā medicīnas ētikas Komiteja
Beth Israel Deaconess Medical Center (BIDMC)	USA	Dana-Farber/Harvard Cancer Center Institutional Review Board
Beckman Research Institute of the City of Hope (BRICOH)	USA	City of Hope Institutional Review Board
Copenhagen Breast Cancer Study (CBCS)	Denmark	De Videnskabsetiske Komiteer I Region Hovedsladen
Spanish National Cancer Centre (CNIO)	Spain	Instituto de Salud Carlos III Comité de Bioética y Bienestar Animal
City of Hope Cancer Center (COH)	USA	City of Hope Institutional Review Board
CONsorzio Studi ITaliani sui Tumori Ereditari Alla Mammella (CONSIT TEAM)	Italy	Comitato Etico Indipendente della Fondazione IRCCS "Istituto Nazionale dei Tumori"
National Centre for Scientific Research Demokritos (DEMOKRITOS)	Greece	Bioethics committee of NCSR ‘‘Demokritos’’
Dana Farber Cancer Institute (DFCI)	USA	Dana-Farber/Harvard Cancer Center Institutional Review Board
Deutsches Krebsforschungszentrum (DKFZ)	Germany	Ethik-Kommission des Klinikums der Universität
(DKFZ - addtional)	Columbia	Hospital Universitario de San Ignacio Comité de Investigaciones y Etica
(DKFZ - addtional)	Pakistan	Shaukat Khanum Memorial Cancer Hospital and Research Centre Institutional Review Board
Epidemiological study of BRCA1 and BRCA2 mutation carriers (EMBRACE)	UK and EIRE	Anglia & Oxford MREC
Fox Chase Cancer Center (FCCC)	USA	Institutional Review Board Fox Chase Cancer Center
Ghent University Hospital (G-FAST)	Belguim	Universitair Ziekenhuis Gent - commissie voor medische ethiek
German Consortium of Hereditary Breast and Ovarian Cancer (GC-HBOC)	Germany	Ethik-Kommission der Medizinischen Fakultät der Universät zu Köln
Genetic Modifiers of cancer risk in BRCA1/2 mutation carriers (GEMO)	France	Comité consultatif sur le traitement de l'information en matière de recherche dans le domaine de la santé

Georgetown University (GEORGETOWN)	USA	MedStar Research Institute - Georgetown University Oncology Institutional Review Board
Hospital Clinico San Carlos (HCSC)	Spain	Comité Ético de Investigación Clínia Hospital Clínico San Carlos
Helsinki Breast Cancer Study (HEBCS)	Finland	Helsingin ja uudenmaan sairaanhoitopiiri (Helsinki University Central Hospital ethics committee)
HEreditary Breast and Ovarian study Netherlands (HEBON)	Netherlands	Protocol Toetsingscommissie van het Nederlands Kanker Instituut/Antoni van Leeuwenhoek Ziekenhuis
Hungarian Breast and Ovarian Cancer Study (HUNBOCS)	Hungary	Institutional Review Board of the Hungarian National Institute of Oncology
Univeristy Hospital Vall d'Hebron (HVH)	Spain	The Hospital Universitario Vall d'Hebron Clinical Research Ethics Committee
Institut Català d'Oncologia (ICO)	Spain	Catalan Institute of Oncology Institutional Review Board
International Hereditary Cancer Centre (IHCC)	Poland	Komisji Bioetycznej Pomorskiej Akademii Medycznej (Pomeranian Medical University Bioethics Committee)
Iceland Landspitali - University Hospital (ILUH)	Iceland	Vísindasiđanefnd National Boethics Committee
Istituto Oncologico Veneto Hereditary Breast and Ovarian Cancer Study (IOVHBOCS)	Italy	Centro Oncologico Regionale Azienda Ospedale Di Padova Comitato Etico
Portuguese Oncology Institute-Porto Breast Cancer Study (IPOBCS)	Portugal	Comissão de Ética para a Saúde (CES) do IPO-Porto
Kathleen Cuningham Foundation Consortium for Research into Familial Breast Cancer (KCONFAB)	Australia	Peter MacCallum Cancer Centre Ethics Committee
(KCONFAB - additional)	Australia	Queensland Institute of Medical Research - Human Research Ethics Committee
University of Kansas Medical Center (KUMC)	USA	University of Kansas Medical Center Human Subjects Committee
Modifiers and Genetics in Cancer (MAGIC)	USA	University of Pennsylvania Institutional Review Board
Mayo Clinic (MAYO)	USA	Mayo Clinic Institutional Review Boards
Memorial Sloane Kettering Cancer Center (MSKCC)	USA	Memorial Sloan-Kettering Cancer Center IRB
(MSKCC - additional)	USA	Human Biospecimen Utilization Committee
General Hospital Vienna (MUV)	Austria	Ethikkommission der Medizinischen Universität Wien
National Cancer Institute (NCI)	USA	NIH Ethics Office
N.N. Petrov Institute of Oncology (NNPIO)	Russia	N.N. Petrov Institional Ethical Committee
NorthShore University HealthSystem (NORTHSHORE)	USA	NorthShore University HealthSystem Institutional Review Board
Ontario Cancer Genetics Network (OCGN)	Canada	Mount Sinai Hospital Research Ethics Board
The Ohio State University Comprehensive Cancer Centre (OSU CCG)	USA	Cancer Institutional Review Board
Odense University Hospital (OUH)	Denmark	Den Videnskabsetiske Komité for Region Syddanmark
Pisa Breast Cancer Study (PBCS)	Italy	Comitato Etico per lo studio del farmaco sull'uomo (Ethics Committee for the study of the drug on humans)
Swedish Breast Cancer Study (SWE-BRCA)	Sweden	Regionala Etikprövningsnämnden Stockholm
University of Chicago (UCHICAGO)	USA	University of Chicago Biological Sciences Division IRB
University of California San Francisco (UCSF)	USA	Committee on Human Research
UK and Gilda Radner Familial Ovarian Cancer Registries (UKGRFOCR)	UK	Cambridge Local Research Ethics Committee

(UKGRFOCR - additional)	USA	Roswell Park Cancer Institute IRB
University of Pennsylvania (UPENN)	USA	University of Pennsylvania Institutional Review Board
Cancer Family Registry at Magee-Womens Hospital (UPITT)	USA	The University of Pittsburgh Institutional Review Board
Victorian Familial Cancer Trials Group (VFCTG)	Australia	Peter MacCallum Cancer Centre Ethics Committee
Women’s Cancer Research Institute (WCP)	USA	Cedars-Sinai Institutional Review Board
