# Supplementary material for: Identification of independent association signals and putative functional variants for breast cancer risk through fine-scale mapping of the 12p11 locus
Source: Breast Cancer Res. 2016 Jun 21;18:64. doi: 10.1186/s13058-016-0718-0 (PMC4962376; doi:10.1186/s13058-016-0718-0)
Supplement: Additional file 2: Table S3. — Independent association signals for risk of estrogen (ER)-positive and ER-negative breast cancer in European descendants. (PDF 47 kb) [file 13058_2016_718_MOESM2_ESM.pdf]

Table S3 Independent association signals for risk of estrogen (ER) -positive and ER-negative breast cancer in European descendants

SNPs*	Position (hg 19)	Alleles	EAF	Univariate Analysis		Conditional Analysis [b]	
				Per-allele OR (95% CI) [a]	*P* trend	Per-allele OR (95% CI) [c]	*P* trend
ER-positive breast cancer [e]							
rs7297051	28174817	T*/C	0.23	0.89 (0.87-0.91)	4×10^{-18}	0.92 (0.89-0.95)	7×10^{-7}
rs805510	28139846	T*/C	0.11	0.87 (0.84-0.90)	3×10^{-15}	0.93 (0.89-0.98)	3×10^{-3}
rs1871152	28379826	G*/A	0.31	0.95 (0.93-0.97)	9×10^{-5}	0.97 (0.94-0.99)	0.01
ER-negative breast cancer [f]							
rs7297051	28174817	T*/C	0.22	0.87 (0.83-0.91)	3×10^{-10}	0.91 (0.86-0.96)	8×10^{-4}
rs805510	28139846	T*/C	0.10	0.83 (0.79-0.88)	8×10^{-10}	0.90 (0.84-0.97)	7×10^{-3}
rs1871152	28379826	G*/A	0.30	0.93 (0.90-0.97)	2×10^{-3}	0.95 (0.91-1.00)	0.03

EAF, effect allele frequency in cases; LD, linkage disequilibrium; OR, odds ratio; CI, confidence interval.

[*] Lead SNPs for the three independent signals identified in the analysis for overall breast cancer risk.

[a] Adjusted for studies, and the top principle components.

[b] Test for heterogeneity between ER-positive and negative groups in the conditional analysis was conducted. The *P*-values for rs7297051, rs805510, and rs1871152 were 0.72, 0.44, and 0.51, respectively.

[c] Included all three variants, and was adjusted for studies, and the top eight principle components.

[d] Using stepwise regression analysis, three independent signals were identified for ER-positive breast cancer: rs7297051, rs805510, and rs7959641(lies in signal 3 and in moderate LD with rs1871152 with an r^2 of 0.28).

[f] Using stepwise regression analysis, three independent signals were identified for ER-negative breast cancer: rs7297051, rs805510, and rs79712894 (lies in signal 3 and in LD with rs1871152 with an r^2 of 0.86).
