# Supplementary material for: Identification of independent association signals and putative functional variants for breast cancer risk through fine-scale mapping of the 12p11 locus
Source: Breast Cancer Res. 2016 Jun 21;18:64. doi: 10.1186/s13058-016-0718-0 (PMC4962376; doi:10.1186/s13058-016-0718-0)
Supplement: Additional file 3: Table S4. — Associations of independent signals for breast cancer risk for BRCA1 mutation carriers. (PDF 64 kb) [file 13058_2016_718_MOESM3_ESM.pdf]

Table S4. Associations of independent signals for breast cancer risk for BRCA1 mutation carriers

Signals	SNPs	Position (hg 19)	Alleles	EAF	LD (r^2) [§]	Univariate Analysis		Conditional Analysis	
						Per-allele HR (95% CI) [a]	*P* trend	Per-allele HR (95% CI) [b]	*P* trend
Index [‡]	rs10771399	28155080	G*/A	0.10	-	0.86 (0.80-0.91)	$3×10^{-6}$	-	-
Top SNPs identified for women of European descents in BCAC studies									
Signal 1	rs7297051	28174817	T*/C	0.23	0.37	0.89 (0.85-0.93)	$3×10^{-7}$	0.94 (0.90-0.98)	0.01
Signal 2	rs805510	28139846	T*/C	0.11	0.85	0.85 (0.80-0.90)	$3×10^{-7}$	0.94 (0.88-1.01)	0.07
Signal 3	rs1871152	28379826	G*/A	0.31	0.04	0.92 (0.88-0.96)	$1×10^{-4}$	0.96 (0.93-1.00)	0.03

EAF, effect allele frequency in all participants; LD, linkage disequilibrium; HR, hazard ratio; CI, confidence interval.

[‡] Identified in the initial GWAS conducted for women of European descent [1].

[*] Effect alleles.

[§] Linkage disequilibrium with rs10771399 for BRCA1 mutation carriers.

[a] Adjusted for studies, and the top principal components.
