# Supplementary material for: Identification of independent association signals and putative functional variants for breast cancer risk through fine-scale mapping of the 12p11 locus
Source: Breast Cancer Res. 2016 Jun 21;18:64. doi: 10.1186/s13058-016-0718-0 (PMC4962376; doi:10.1186/s13058-016-0718-0)
Supplement: Additional file 4: Table S5. — Associations of independent signals for breast cancer risk in women of East Asian and African descent. (PDF 66 kb) [file 13058_2016_718_MOESM4_ESM.pdf]

Table S5 Associations of independent signals for breast cancer risk in women of East Asian and African descent

Signal	SNPs	Position (hg 19)	Allele	EAF	LD[§] (R^2)	Univariate Analysis	
						Per-allele OR (95% CI) [b]	*P* trend [b]
**Women of East Asian ancestry**							
Index [‡]	rs10771399	28155080	G*/A	0.17	-	0.87 (0.81-0.93)	5×10^{-5}
Signal 1	rs7297051	28174817	T*/C	0.22	0.70	0.88 (1.07-1.21)	2×10^{-5}
Signal 2	rs805510	28139846	T*/C	0.15	0.86	0.88 (0.82-0.94)	5×10^{-4}
Signal 3	rs1871152	28379826	G*/A	0.08	0.00	0.96 (0.86-1.07)	0.48
Signal 4	rs113824616 [a]	28184905	C*/T	0.08	0.62	0.84 (0.70-0.99)	0.04
**Women of African ancestry**							
Index [‡]	rs10771399	28155080	G*/A	0.04	-	0.82 (0.58-1.14)	0.23
Signal 1	rs7297051	28174817	T*/C	0.13	0.14	0.95 (0.79-1.14)	0.61
Signal 2	rs805510	28139846	T*/C	0.45	0.01	0.96 (0.85-1.08)	0.51
Signal 3	rs1871152	28379826	G*/A	0.37	0.00	0.95 (0.83-1.08)	0.47
Signal 4	rs113824616 [a]	28184905	C*/T	0.01	0.56	0.86 (0.12-5.70)	0.87

EAF, effect allele frequency in controls; LD, linkage disequilibrium; OR, odds ratio; CI, confidence interval.

[‡] Identified in the initial GWAS conducted in women of European descent (1).

[*]Effect alleles.

[§] Linkage disequilibrium with rs10771399 in women of Asian or African descent.

[a] The OR for rs113824616 were estimated using ER(-) cases and all controls.

[b] Adjusted for studies, and the top two principal components.
