# Supplementary material for: Identification of independent association signals and putative functional variants for breast cancer risk through fine-scale mapping of the 12p11 locus
Source: Breast Cancer Res. 2016 Jun 21;18:64. doi: 10.1186/s13058-016-0718-0 (PMC4962376; doi:10.1186/s13058-016-0718-0)
Supplement: Additional file 5: Table S2. — List of the variants that were retained for further functional annotation in European descendants. (PDF 54 kb) [file 13058_2016_718_MOESM5_ESM.pdf]

Table S2 List of the variants that were retained for further functional annotation

**Signal 1**	**Signal 2**	**Signal 3**						**Signal 4**
rs812020	Chr12:28140407I	rs11049361	rs1824767	chr12:28346793:D	rs10771406	rs11049511	rs11049606	rs113824616
Chr12:28164044I	rs788458	rs11049416	rs10843135	rs11610011	rs11049391	rs11049527	rs9669509	rs150206154
rs2590275	rs1314085	rs79712894	rs1824766	rs11519332	rs11049491	rs11049417	rs11049620	
rs2619434	Chr12:28144690D	rs11049386	rs7973241	rs11049469	rs10771410	rs10843154	rs11049629	
	Chr12:28140399I	rs10843139	rs11049460	rs11049437	rs10843114	chr12:28451670:D	chr12:28544983:D	
	rs1267210	rs11524516	rs7973656	rs7303747	rs11049496	chr12:28489383:D	rs10506032	
	rs10843068	rs61922969	rs7973516	rs10843141	chr12:28297884:I	rs11049528		
	rs1267212	rs56318627	rs11049442	rs12320545	rs10843103	chr12:28441430:D		
	rs16932559	rs11049476	rs10843133	rs11049370	rs11049566	rs10843172		
	rs788462	rs7974904	rs1585682	rs11049369	rs11049424	rs11049615		
	rs251952	rs7961407	rs61920243	rs10843134	rs11049425	chr12:28604973:I		
	rs10843069	rs11049415	rs11049444	rs11049390	rs11049570	chr12:28480090:D		
	rs11612977	rs10843126	rs11049445	rs11049388	chr12:28297881:I	rs11049594		
	rs1270113	rs10843127	rs34378873	rs17801442	rs11049371	rs7313833		
	rs12231591	rs11049448	rs11049440	rs11049384	rs10771408	rs11049572		
	rs1267205	rs61920242	rs11049441	rs10843140	rs11049493	rs12368745		
	rs10843067	rs11049409	rs60193273	rs11049447	rs11049582	rs7137557		
	rs788464	chr12:28335056:I	rs11049439	rs11049514	rs2045887	rs10843163		
	rs11049293	rs11049414	rs7961769	chr12:28384630:D	rs61920563	rs59193275		
	rs251955	rs9766606	rs7974701	rs11049472	rs10771411	rs59879751		
	rs11049272	rs61920228	rs11049420	rs11049468	rs11049584	rs60960895		
	rs1267206	rs7138173	rs10843123	rs11049474	rs74515560	rs11049577		
	rs2737448	chr12:28337992:I	rs12146881	rs11049467	rs11049495	rs11049537		
	rs10843071	chr12:28337721:D	rs11049446	rs12370271	rs7956671	rs2348235		
	rs251951	rs7307078	rs10843124	rs11049465	rs10843146	rs11049619		
	rs251950	rs12372073	rs10843130	rs7980441	chr12:28361249:I	rs10843167		
	Chr12:28072287	rs12372059	rs11049405	rs10843104	rs11049517	rs10843170		
	rs2737447	chr12:28345826:I	rs11049457	rs1824768	chr12:28624623:D	rs10843169		
	rs805515	rs7974979	rs61920245	rs11049454	rs117066882	rs11049575		
	rs11049292	rs10843137	rs11049455	rs11049376	rs12371059	rs12372448		
	rs1267208	rs1478335	rs73261702	rs12367188	chr12:28432808:D	rs11049581		
	rs10843066	rs1478334	rs61920253	chr12:28343675:D	rs7964793	rs11049426		
	rs788457	rs1478336	chr12:28355420:I	rs11049410	rs2172299	rs11049427		
	rs142450317	rs11049429	rs11049453	rs10219470	rs11049503	rs11049579		
	rs1267213	rs4284426	rs11049480	rs7955118	rs61922974	rs11049612		
	rs788455	chr12:28347065:D	rs10843125	rs7955237	chr12:28312811:I	rs11049618		
	rs11049290	rs11049413	rs7957382	rs11049363	rs61922976	rs11049613		
	rs788463	rs12366932	rs7957503	rs17801436	rs61922975	chr12:28596922:D		

	rs809291	rs11049428	rs4554927	rs11049360	rs7294486	rs12368409
	rs2619433	rs2061758	rs7957059	rs7305286	rs11049666	rs10843162
	rs788459	rs10843138	rs4573721	rs11049470	rs11049430	rs11049545
	rs10843061	rs11049412	rs1551986	rs12371462	rs61922977	rs10843155
	rs2619411	rs11049475	rs1551987	chr12:28316338:D	rs11049667	rs11049538
	rs805513	rs6487671	rs11049402	rs7958401	chr12:28453440:I	rs11049583
	rs10843057	rs11049419	rs10492368	rs11049403	rs11049505	rs10843164
	rs10843058	rs7298652	rs10843129	rs10492369	rs11049507	rs11049547
	rs11049285	rs11049423	chr12:28326163:D	rs11049393	rs11049509	chr12:28333106:D
	rs11049276	rs11049422	rs10843128	rs11049385	rs11049510	chr12:28489862:I
	rs1975930	rs6487669	rs1841962	rs7959641	rs9645730	rs11049540
	rs2737455	chr12:28363949:D	rs7957056	chr12:28369597:D	rs61922978	rs11049604
	rs10771399	rs11049471	rs11612143	chr12:28369596:D	rs11049532	rs61920565
	rs11049280	chr12:28349282:I	chr12:28362760:I	chr12:28276960:D	rs17432330	rs11049543
	rs11049281	rs10843131	rs11049389	rs11049533	rs11049515	rs11049541
	rs11049289	rs11513466	chr12:28329536:D	rs11049576	rs11049516	rs11049544
	rs11049282	rs11049421	rs7969582	rs11049387	rs11049531	rs11049609
	rs11049278	rs11519331	rs7955094	chr12:28276967:I	rs10506029	rs11049608
	rs11049279	rs61920244	chr12:28349087:I	rs10843110	rs11049530	rs11049563
	rs11049283	rs11513467	rs7961395	rs61920241	rs10843150	rs10843159
	rs7976725	rs11049464	chr12:28348381:I	rs4357727	rs61922979	rs11049600
	rs10843053	rs7306838	rs7974882	rs2061760	rs11049521	rs10843157
	rs11049277	rs11494779	rs12368652	rs12369144	rs11049518	rs11049558
	rs10843054	rs11049438	rs11049395	rs11049359	rs10843153	rs11049550
	rs12230582	chr12:28392506:D	rs11049392	rs11049484	rs10843152	rs11049556
	Chr12:28166131I	rs11519330	rs11049394	rs11519095	rs10843151	rs61920564
	Chr12:28060602	rs11513250	rs11049399	rs1479492	rs11049520	rs11049555
	rs809816	rs11513465	rs61920227	rs11049485	rs3782517	rs12371222
	rs10843064	rs11519094	rs11049397	rs10843143	rs3825246	rs11049567
	rs10843063	rs7956418	rs11049400	rs11049488	rs3782515	rs11049554
	rs11049295	rs7971033	rs11049401	rs10771409	rs11049524	rs10843158
	rs11049286	rs11049443	rs10843132	rs57618147	rs11049525	rs11049617
	rs1838564	rs9645745	rs12372372	rs10843111	rs12371974	rs11049552
	rs2619415	rs11049407	rs11049478	rs10843112	rs11049526	rs11049553
	rs10843062	rs11049459	chr12:28300276:I	rs716687	rs184460060	rs61915974
	rs10843059	rs11049458	rs11049398	rs7980151	rs60703205	rs11049549
