# Supplementary material for: Identification of independent association signals and putative functional variants for breast cancer risk through fine-scale mapping of the 12p11 locus
Source: Breast Cancer Res. 2016 Jun 21;18:64. doi: 10.1186/s13058-016-0718-0 (PMC4962376; doi:10.1186/s13058-016-0718-0)
Supplement: Additional file 6: Table S6. — Putative functional SNPs identified using the ENCODE data. (PDF 50 kb) [file 13058_2016_718_MOESM6_ESM.pdf]

Table S6 Putative functional SNPs identified using the ENCODE data

Signal	SNP	Position	Alleles	Imputation R^2	European descendants		East Asian descendants		African descendants		RegulomeDB score [a]
					OR 95% CI	*P*-value	OR 95% CI	*P*-value	OR 95% CI	*P*-value	
1	rs812020	28151609	C*/A	Genotyped	0.89 (0.87-0.91)	2E-27	0.88 (0.83-0.94)	5E-05	0.93 (0.82-1.06)	0.27	3a
2	rs788463	28152648	A*/T	Genotyped	1.17 (1.14-1.21)	5E-25	1.16 (1.08-1.24)	3E-05	0.93 (0.61-1.41)	0.72	2b
2	rs10843066	28176797	T*/C	Genotyped	1.17 (1.14-1.21)	3E-25	1.14 (1.07-1.22)	8E-05	1.00 (0.76-1.31)	0.97	2b
3	rs10843110	28289141	T*/G	0.999	1.06 (1.04-1.08)	2E-07	0.95 (0.87-1.03)	0.21	1.09 (0.95-1.24)	0.22	2b
3	rs56318627	28345652	C*/T	0.9995	1.06 (1.04-1.08)	6E-08	1.06 (0.94-1.19)	0.35	1.04 (0.91-1.19)	0.57	2b
3	rs11049453	28355929	G*/A	Genotyped	1.06 (1.04-1.08)	9E-08	1.06 (0.94-1.19)	0.32	1.03 (0.88-1.19)	0.71	2b

[*] Effect allele.

[a] A Regulome DB score of 2b suggests that the SNP is predicted to be likely to affect gene expression level, and the evidence includes transcription factor binding, any motif change, DNase Footprint, and DNase peak; a score of 3a suggest that the evidence for the SNP is from transcription factor binding, any motif change, and DNase peak.
