# Supplementary material for: Identification of independent association signals and putative functional variants for breast cancer risk through fine-scale mapping of the 12p11 locus
Source: Breast Cancer Res. 2016 Jun 21;18:64. doi: 10.1186/s13058-016-0718-0 (PMC4962376; doi:10.1186/s13058-016-0718-0)
Supplement: Additional file 7: Table S7. — Gene expression analysis for putative functional SNPs using 1,006 breast tumor samples in TCGA. (PDF 46 kb) [file 13058_2016_718_MOESM7_ESM.pdf]

Table S7 Gene expression analysis for putative functional SNPs using 1,006 breast tumor samples in TCGA

SNP ID	Gene	Effect Allele	Ref Allele	Beta [a]	*P*-value
**Signal 1**					
rs812020	*PTHLH*	A	C	0.03	0.65
rs812020	*CCDC91*	A	C	0.06	0.27
**Signal 2**					
rs788463	*PTHLH*	A	T	0.04	0.69
rs10843066	*PTHLH*	T	C	0.07	0.41
rs788463	*CCDC91*	A	T	0.01	0.88
rs10843066	*CCDC91*	T	C	0.03	0.70
**Signal 3**					
rs10843110	*PTHLH*	T	G	0.13	0.02
rs56318627	*PTHLH*	C	T	0.15	0.01
rs11049453	*PTHLH*	G	A	0.15	0.01
rs10843110	*CCDC91*	T	G	-0.11	0.08
rs56318627	*CCDC91*	C	T	-0.11	0.07
rs11049453	*CCDC91*	G	A	-0.13	0.03

[a] The regression coefficient for each SNP estimated in the residual linear regression.
